# Simulating Heterogeneous Tumor Cell Populations

**DOI:** 10.1371/journal.pone.0168984

**Published:** 2016-12-28

**Authors:** Andrew Sundstrom, Dafna Bar-Sagi, Bud Mishra

**Affiliations:** 1 Department of Pharmacology and Systems Therapeutics, Icahn School of Medicine at Mount Sinai, New York, NY, United States of America; 2 Department of Biochemistry and Molecular Pharmacology, NYU School of Medicine, New York, NY, United States of America; 3 Department of Computer Science, Courant Institute of Mathematical Sciences, New York, NY, United States of America; Universidade de Sao Paulo, BRAZIL

## Abstract

Certain tumor phenomena, like metabolic heterogeneity and local stable regions of chronic hypoxia, signify a tumor’s resistance to therapy. Although recent research has shed light on the *intracellular* mechanisms of cancer metabolic reprogramming, little is known about how *tumors* become metabolically heterogeneous or chronically hypoxic, namely the initial conditions and spatiotemporal dynamics that drive these cell population conditions. To study these aspects, we developed a minimal, spatially-resolved simulation framework for modeling tissue-scale mixed populations of cells based on diffusible particles the cells consume and release, the concentrations of which determine their behavior in arbitrarily complex ways, and on stochastic reproduction. We simulate cell populations that self-sort to facilitate metabolic symbiosis, that grow according to tumor-stroma signaling patterns, and that give rise to stable local regions of chronic hypoxia near blood vessels. We raise two novel questions in the context of these results: (1) How will two metabolically symbiotic cell subpopulations self-sort in the presence of glucose, oxygen, and lactate gradients? We observe a robust pattern of alternating striations. (2) What is the proper time scale to observe stable local regions of chronic hypoxia? We observe the stability is a function of the balance of three factors related to *O*_2_—diffusion rate, local vessel release rate, and viable and hypoxic tumor cell consumption rate. We anticipate our simulation framework will help researchers design better experiments and generate novel hypotheses to better understand dynamic, emergent whole-tumor behavior.

## Introduction

Tumors are disorganized, heterogeneous tissues, consisting of many distinct cell types in spatially complex arrangements. The somatic evolution of early carcinogenesis feeds on sources of phenotypical variation present in tumor and surrounding stromal tissue. Besides the polyclonal proliferating cancer cell population undergoing somatic evolution [1], tumors include non-proliferating stromal cells, fibroblasts, immune cells, extracellular matrix, collagen, blood vessels, and other structures and cell types [2–4]—these include “normal” cells that are conscripted by transformed cells to play collaborative roles in the neoplastic agenda. So there is a large degree of genotypic and phenotypic heterogeneity composing a tumor. Since tumors originate in physiological structures that range from simple epithelial sheets to ducts to neural and muscle tissue, and often invade neighboring tissues and then metastasize, colonizing distant tissues, the spatial situations and geometric structural relationships of tumors are themselves complex and heterogeneous. Add to this the dynamic character of the microenvironment, from high frequency variations in oxygen, nutrient, and signaling molecule concentrations, to longer time scale processes like the synthesis of extracellular matrix and blood vessels during angiogenesis.

As a tumor grows, it rapidly outstrips its blood supply. High proliferation causes high cell density that overtaxes local oxygen supply. This leaves portions of the tumor with an oxygen concentration significantly lower than in healthy tissues. This stress condition is tumor hypoxia. Hypoxia is strongly correlated with poor prognosis as it renders tumors less responsive to chemotherapy and radiotherapy [4–6]. Hypoxia-inducible factors (HIFs) are transcription factors that respond to changes in available oxygen in the cellular environment, specifically to hypoxia. When activated, HIF-1 upregulates several genes to promote survival in low-oxygen conditions. These include glycolysis enzymes that allow cells to synthesize ATP in an oxygen-independent manner, and vascular endothelial growth factor (VEGF) that cells release to promote angiogenesis. So hypoxia is directly instrumental in tumor progression. Prolonged or extreme hypoxia can lead to necrosis, and tumors often have central regions called necrotic cores [7]. Necrosis in turn activates inflammatory responses that produce cytokines that stimulate tumor growth [4]. Recent research has investigated the interactions between hypoxic tumor cells and immune cells (tumor-associated macrophages [8]) and cells that synthesize extracellular matrix (tumor-associated fibroblasts [9, 10]). Both are involved with inflammatory processes tied to tumor progression. In the context of the tumor microenvironment, these interactions regulate tumor properties like spatial patterns of cell localization, angiogenesis, and collective invasion and migration [11, 12]. Thus it is of theoretical and clinical significance to understand how, and under what conditions, hypoxia arises in tumors.

We are especially interested in simulating how tumor chronic hypoxia interacts with tumor metabolic heterogeneity [13–15]. In tumors, while the Warburg effect [16, 17] is commonly observed, aerobic glycolysis is not the only metabolic program cancer cells follow. In fact, there are experiments and mathematical models to suggest the metabolic strategies tumors use, when considered as a whole, are quite dynamic [18–20]. As a tumor progresses, it negotiates a course of barriers to proliferation [2, 3, 21]. Its gain of oncogenic function [22–29], and loss of tumor suppressor function [30–32], give rise to changing bioenergetic and biosynthetic requirements for proliferation in the face of the new obstacles [20]. So the tumor cells’ metabolic programs are varied and in flux, following from this coevolution. Direct *in vivo* detection and quantification of metabolic heterogeneity dates back to at least 1990, using ^1^H NMR spectroscopic imaging of human intracranial tumors [33], and has progressed more recently to use 3D high-resolution fluorescence imaging, for example, of human breast tumors [34], and ^18^F-FDG PET/CT imaging, for example, of invasive ductal carcinoma of the breast [35] and head and neck tumors [36]. One recent study finds a hypoxic non-Warburg metabolic phenotype plays an active role in tumorigenesis [37].

For a long time, there was no systematic characterization of metabolic pathways active in transformed cells, so the contribution of these pathways in promoting rapid cancer cell proliferation was unclear. But in 2012, Jain, *et al* [38] produced a comprehensive metabolite profile for each of the NCI-60, a set of sixty well-characterized primary human cancer cell lines established from nine common tumor types. To systematically characterize cancer cell metabolism, they created cellular consumption and release (CORE) profiles of 219 metabolites spanning the major pathways of intermediate metabolism. We were particularly encouraged by the conceptual approach Jain, *et al* undertook in their methods, namely, that cancer cell metabolic reprogramming *manifests* as altered nutrient uptake and release. In other words, the gross quantitative properties of cellular consumption rate and release rate of metabolites (and other particles, like gasses and signaling molecules) is sufficient to characterize and distinguish cancer cell metabolic phenotypes from an extracellular perspective.

When one is deciding how to create a computational model of a cell population, the primary choice is whether it should be continuous or discrete (or a hybrid). Usually, this breaks out into two canonical design dimensions. In the first, one can represent cells as points, or as being composed of sub-elements. In the second, cells can occupy positions on a fixed, regular lattice, or positions off-lattice. The lattice-gas cellular automata model [39] is an example of cells-as-points on a lattice. The off-lattice hybrid discrete-continuum model [40] is an example of cells-as-points off-lattice. The cellular Potts model [41–49] is an example of cells composed of sub-elements on a lattice. The sub-cellular viscoelastic model [50] is an example of cells composed as sub-elements off-lattice. Considering the canonical modeling dimensions, one should weigh several trade-offs. When one models cells as points, one can represent large numbers of cells in a computationally efficient manner; but the model is coarse-grained, neglecting cell mechanics and other biophysical considerations. When one models cells composed of sub-elements, one can better represent cell shape, cytoskeleton, and internal structure; but this requires more computation and one can therefore represent fewer objects. Lattice-based models are computationally efficient and afford a simpler algorithmic design; but the complexity depends on lattice size, not the number of objects, and the rigid structure of the lattice can affect morphology and behavior. Off-lattice models have a complexity that depends on the number of objects being modeled, and one can model cell movement and morphology continuously; but collision detection is computationally expensive, and interactions between nearby elements can be more expensive than using a lattice.

We restrict our focus to individual-based, spatially-resolved, diffusive models that can represent the gross metabolic phenotypical properties measured by Jain, *et al* [38], namely distinct consumption and release profiles, and particle types with distinct diffusion rates. Using the spatial simulation framework developed by Cleveland, *et al* [51] as a starting point, we developed a fast, minimal simulation of a metabolically and spatially heterogeneous cell population with diffusible metabolites, gasses, and signaling molecules. We extend the framework in significant and novel ways to further suit our needs. First, we can support any number of cell types and any number of particle types (each with its own diffusion rate). Second, each cell type has default behaviors, as before, and conditional behaviors, which can implement phenotypical adaptations and mutations, and state machines composed of two or more cell types. Third, initial, and upper- and lower-bounded basal concentrations can be set for each particle type. Fourth, each cell type can be replaceable or not, and reproductive or not. Fifth, initial lattice occupation can be delayed to establish complex diffusion gradients to form prior to simulation.

We observe important emergent phenomena such as metabolic symbiosis [52, 53], necrotic core formation, and stable local regions of chronic hypoxia like we observe in xenographed hypoxic tumor histology (see the Materials and Methods section below for histology image availability). The 3D lattice data structure is simple, regular, and easy to interrogate by feature measurer and feature integrator modules one can later implement to detect emergent phenomena—we elaborate on this in the conclusion.

## Materials and Methods

### Cellular automaton

The universe of the simulation is a 2D or 3D cellular automaton [54]. Hereafter, for the sake of defining the simulation, we shall assume a 3D configuration, though many of the examples appearing on the page will naturally lend themselves better to a 2D presentation. A cellular automaton works according to the following principles. Each box inside a regular 3D lattice represents a cell, whose position is specified by an integer-valued three-tuple, (*i*, *j*, *k*). Each cell is one of a finite number of states. For simplicity, let us assume these states are “on” and “off.” Each cell has a well-defined neighborhood of adjacent cells, where neighborhood can be defined in a flexible way, most commonly immediate neighbors. All cells are initialized to some state at time = 0. Then, at each subsequent time step (time = 1, 2, …), the cells’ states are updated according to fixed rules that determine the new state of the cell as a function of the cell’s current state and those of its neighbors. The state update rules are applied uniformly and simultaneously to the whole lattice of cells. In this way, the cellular automaton evolves as a whole, and patterns in the population of cells emerge over time that cannot be predicted without performing the requisite computation.

### The spatial simulation

In the context of simulating large, heterogeneous cell populations, we need a flexible and extensible framework. We first specify the dimensions of our universe as *x_dim*, *y_dim*, and *z_dim* parameters—running examples will execute on a 40 × 40 × 40 lattice—and the total running time of the simulator, where time steps are in arbitrary time units, and can be scaled using a time parameter, *τ*. We wish to simulate *M* types of cell: *T* = {*v*, *ε*, *α*, *β*, *γ*, *δ*, …}, where *v* and *ε* are special types related to a blood vessel and “empty” space, respectively. (Note “empty” may be simply interpreted as “devoid of any cellular function”; units of “empty” space could be used to model a lumen, the inside space of a tubular structure, which is of particular interest to those modeling colonic crypts, or lung and pancreatic adenocarcinomas, for instance.) We further wish to simulate *N* types of diffusible particles that cells can consume and release. At any given time, each (*i*, *j*, *k*) is occupied by some *t* ∈ *T*. Each cell type, *t* ∈ *T*∖{*v*, *ε*}, has a two sets of parameters that specify its behavior.

First are the default parameters. These include: *c*_*t*,*p*_, the consumption rate of particle type *p*; *r*_*t*,*p*_, the release rate of particle type *p*; *σ*_*t*,*p*_, the impact factor of each particle type *p*; whether or not *t* is replaceable; and whether or not *t* is reproductive. These can be thought of as implementing the cells’ genotypical (native) behaviors, as viewed from an outside, cell population perspective.

Second are the conditional parameters. These are formulated as trigger-action pairs. A trigger is a set of one or more predicates that are based on particle concentrations, as measured by the cell in its locality. All trigger predicates must be true to execute the associated action. An action is a set of commands which are executed sequentially. The possible commands include: apoptosis; become (“jump to”) another cell type, *t*′ ∈ *T*∖{*v*, *ε*, *t*}; set the consumption rate of particle type *p* to a target value; set the release rate of particle type *p* to a target value; set the impact factor of particle type *p* to a target value; set the Boolean condition of being replaceable; and set the Boolean condition of being reproductive. This conditional degree of flexibility grants us the ability to implement cell mutational events (the action to become another cell type) and the cells’ phenotypical response behaviors (all of the actions), again as viewed from an outside, cell population perspective.

The initial cell population in the lattice constitutes another set of parameters. The default cell type for the lattice is empty (*ε*). One can manually specify cell types at individual locations, or can algorithmically do this, using arbitrary functions, including stochastic ones, to specify the initial population. An initialization delay parameter specifies when the all-empty lattice is replaced by its initial configuration. Its default value is 0, but can be set to any future time step, to allow, for example, one to establish a concentration gradient (or set of gradients), that may take many time steps, prior to introducing the cells into it.

Each time step drives the simulation through four phases. Particles are consumed and released according to the cell type’s consumption and release rates, respectively, for that particle type. Particles continually diffuse in $R^{3}$ according to the particle type’s diffusion rate. A cell’s fitness is first individually computed as a function of impinging concentrations of the particles in combination with the cell type’s impact factors corresponding to each particle type. Then its fitness is computed from the fitness scores of individuals in its neighborhood, according to their cell types. This defines a distribution that will be statistically sampled to determine what cell type each lattice location will contain in the next time step.

### Phase 1: consume and release particles

Let *P* denote the set of particle types, and *ρ*_*p*_(*i*, *j*, *k*) denote the normalized concentration of particle type *p* ∈ *P* at location (*i*, *j*, *k*). Concentrations of particles evolve at each time step by the following. For each particle type *p* ∈ *P*, for each cell type *t* ∈ *T*∖{*v*, *ε*}, set

$$\begin{array}{c} \rho_{p}\left(i,j,k\right)=\rho_{p}\left(i,j,k\right)\cdot \left(1-c_{t,p}+r_{t,p}\right). \end{array}$$ (1)

Note that cell type *v* (vessel) has some special default properties related to particles. These defaults implement an assumption we make that a vessel is a perfect source for certain particles (releasing them to full concentration) and a perfect sink for others (consuming them to zero concentration). That is, if location (*i*, *j*, *k*) contains cell type *v*, then ∀*τ*: ∀*p* ∈ *P*, *r*_*v*,*p*_ = 1: *ρ*_*p*_(*i*, *j*, *k*) = 1 and ∀*τ*: ∀*p* ∈ *P*, *c*_*v*,*p*_ = 1: *ρ*_*p*_(*i*, *j*, *k*) = 0. These default properties can be overridden by specifying non-unity vessel consumption and release rates for each particle type. In addition, should one wish to simulate more complex scenarios involving vessels—like the heterogeneity of perfusion—one can define a family of new cell types that act like vessels, each of which has distinct consumption and release rates, and populate the regions of interest accordingly.

The simulator has additional parameters related to particle concentrations. Initial concentrations, and basal upper and lower bounds, can be set for each particle type. For the latter, the simulator enforces the bounds in this phase at each time step: those concentrations falling below the lower bound are set to the lower bound; those rising above the upper bound are set to the upper bound.

### Phase 2: diffuse particles

At each time step, each lattice point’s concentration of the particles it contains is updated according to the diffusion rate, *D*_*p*_, of each particle type *p*. Each particle type’s concentration field, *ρ*_*p*_, undergoes an isotropic 3D Gaussian convolution, using a 5 × 5 × 5 mask with $\sigma =\sqrt{2D_{p}}$. The *n*-dimensional Gaussian kernel is defined as

$$\begin{array}{c} G_{n}\left(\vec{x},\sigma \right)=\frac{1}{{\left(\sqrt{(}2\pi )\sigma \right)}^{n}}e^{-\frac{{|\vec{x}|}^{2}}{2\sigma^{2}}}. \end{array}$$ (2)

We assume input array values outside the bounds of the array are equal to the nearest array border value. We accomplish the convolution using Matlab’s imfilter command.

Equivalently, consider each individual particle taking a random walk in each of the three dimensions [55]. Let *q* be a number drawn from a standard normal distribution, *q* ∼ *N*(0, 1), and *τ* be a scaled time variable with respect to the simulation’s clock tick value. Concentrations of particles diffuse at each time step by the following. For each particle *p*′ of type *p* ∈ *P*,

$$\begin{array}{c} \Delta x_{p^{\prime }}=\Delta y_{p^{\prime }}=\Delta z_{p^{\prime }}=q\sqrt{2D_{p}\tau }. \end{array}$$ (3)

### Phase 3: compute fitness scores

For each location in the lattice (*i*, *j*, *k*), and for each cell type *t* ∈ *T*∖{*v*, *ε*}, let us define a local fitness function

$$\begin{array}{c} f_{t}\left(i,j,k\right)=\left[\sigma_{t,1}\cdot \rho_{1}\left(i,j,k\right)+\sigma_{t,2}\cdot \rho_{2}\left(i,j,k\right)+...+\sigma_{t,n}\cdot \rho_{n}\left(i,j,k\right)\right]\cdot I_{t}, \end{array}$$ (4)

where *ρ*_*p*_(*i*, *j*, *k*) denotes the normalized concentration of particle type *p* at location (*i*, *j*, *k*); *σ*_*t*,*p*_ denotes the impact of particle type *p* on *t*; and $I_{t}$ denotes the indicator function that is *true* if and only if location (*i*, *j*, *k*) is occupied by cell type *t*, reflecting the simulator design assertion of exclusive occupancy of one cell type per location. We define *f*_*ε*_(*i*, *j*, *k*) = 0.

After we compute these individual fitness scores for each lattice location, we use them to decide probabilistically what cell type each location (*i*, *j*, *k*) should contain in the next time step. For each location in the lattice (*i*, *j*, *k*), and for each cell type *t* ∈ *T*∖{*v*}, let us define a neighborhood fitness function

$$\begin{array}{c} F_{t}\left(i,j,k\right)=\frac{1}{N}\sum_{(i^{\prime },j^{\prime },k^{\prime })\in neighbors}f_{t}\left(i^{\prime },j^{\prime },k^{\prime }\right), \end{array}$$ (5)

where *F*_*t*_(*i*, *j*, *k*) denotes the probability that the cell at location (*i*, *j*, *k*) becomes cell type *t*; and *N* denotes the number of neighbors in the sum. Note that since (*i*, *j*, *k*) may reside on an edge or corner of the lattice, the number of its immediate neighbors is bounded from above by 8 (in 2D) and 26 (in 3D).

### Phase 4: reproduce probabilistically

Each cell’s immediate neighbors give a distribution from which to draw the target cell type. Let *S*(*i*, *j*, *k*) = ∑_*t* ∈ *T*∖{*v*}_ *F*_*t*_(*i*, *j*, *k*). In this scheme, if *S*(*i*, *j*, *k*)>1, then we normalize it to 1; and if *S*(*i*, *j*, *k*)<1 then there is some probability that location (*i*, *j*, *k*) will become empty (*t* = *ε*) in the next time step.

We accomplish this as follows. Let shrinkage factor $\lambda =\frac{1}{S(i,j,k)}$. For each *t* ∈ *T*∖{*v*}, shrink each cell type’s probability contribution: *F*_*t*_(*i*, *j*, *k*) = *λ* ⋅ *F*_*t*_(*i*, *j*, *k*). We can represent each *F*_*t*_(*i*, *j*, *k*) as a subinterval of the unit interval. We place them side by side to cover the unit interval. Then we draw a random number, *r*, uniformly in [0, 1]; whichever cell type’s interval *r* resides in determines the target cell type of (*i*, *j*, *k*) for the next time step.

Note that cell type *v* (vessel) defaults to being neither replaceable (its fate is exempt) nor reproductive (it casts no vote), so it is effectively neutral with respect to this reproduction phase of the simulation. Vessels are static features of the spatiotemporal landscape.

### Computing and plotting statistics

Between phases 3 and 4, the simulator computes and displays a number of useful statistics for the user. These are organized into a console style grid of plots below. Since we can often get a good sense of what is happening by examining 2D slices of our 3D world, and because rendering 3D plots is computational expensive, the dashboard consists of mostly 2D plots, and defaults to showing 2D slices on the $\frac{z\_dim}{2}$ plane; for a 40 × 40 × 40 simulation, for example, the 2D plots show the plane *z* = 20.

On the *first* row, in column:

1. Spatial organization of all cell types: 2D slice, color coded
2. Time series plot of the wall clock seconds elapsed at each time step (performance diagnostic)

On the *second* row, in column:

1. Spatial organization of individual fitness of cell type 1: 3D scatter plot, color coded, intensity level denotes fitness
2. same as above for cell types 2, …, *M* − 1
3. Spatial organization of individual fitness of cell type *M*: 3D scatter plot, color coded, intensity level denotes fitness
4. Time series plot of each cell type’s population at each time step, color coded

On the *third* row, in column:

1. Spatial organization of individual fitness of cell type 1: 2D slice, color coded, intensity level denotes fitness
2. same as above for cell types 2, …, *M* − 1
3. Spatial organization of individual fitness of cell type *M*: 2D slice, color coded, intensity level denotes fitness
4. Time series plot of each cell type’s mean individual fitness (± standard deviation) at each time step, color coded

On the *fourth* row, in column:

1. Spatial organization of neighborhood fitness of cell type 1: 2D slice, color coded, intensity level denotes fitness
2. same as above for cell types 2, …, *M* − 1
3. Spatial organization of neighborhood fitness of cell type *M*: 2D slice, color coded, intensity level denotes fitness
4. Time series plot of each cell type’s mean neighborhood fitness (± standard deviation) at each time step, color coded

On the *fifth* row, in column:

1. Time series plot of cell type 1’s *x*, *y*, *z* extents, color coded
2. same as above for cell types 2, …, *M* − 1
3. Time series plot of cell type *M*’s *x*, *y*, *z* extents, color coded
4. Time series plot of each cell type’s whole-image Euler-Poincare characteristic at each time step, color coded

On the *sixth* row, in column:

1. Particle type 1 concentration: 2D slice, mesh plot
2. same as above for particle types 2, …, *N* − 1
3. Particle type *N* concentration: 2D slice, mesh plot

**Fig 1:**
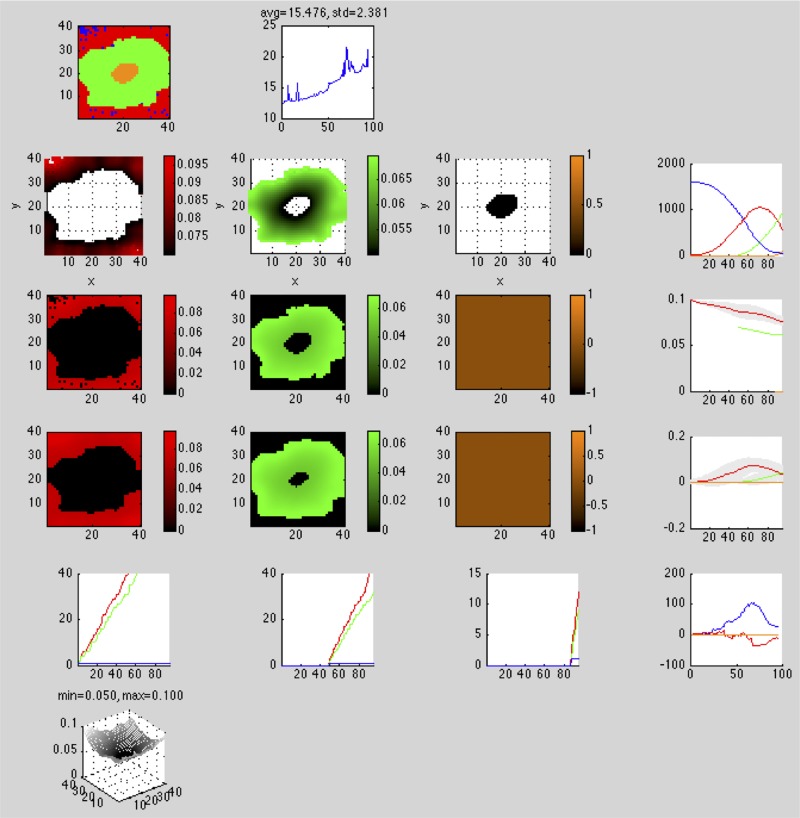
2D simulation console. Simulator console displaying an evolving necrotic core in a 40 × 40 lattice. The simulation consists of four cell types—*empty* (blue), *viable* (red), *hypoxic* (green), and *necrotic* (orange)—and one particle type, *O*_2_. In row 1, column 1, the cell population at this point in the simulation consists of all cell types. In rows 2-5, columns 1-3, all cell types except *empty* have fitness and other spatial statistics reported. In row 6, the concentration of *O*_2_ is reported. In column 4, rows 2-5, aggregate cell type statistics are reported in time series.

**Fig 2:**
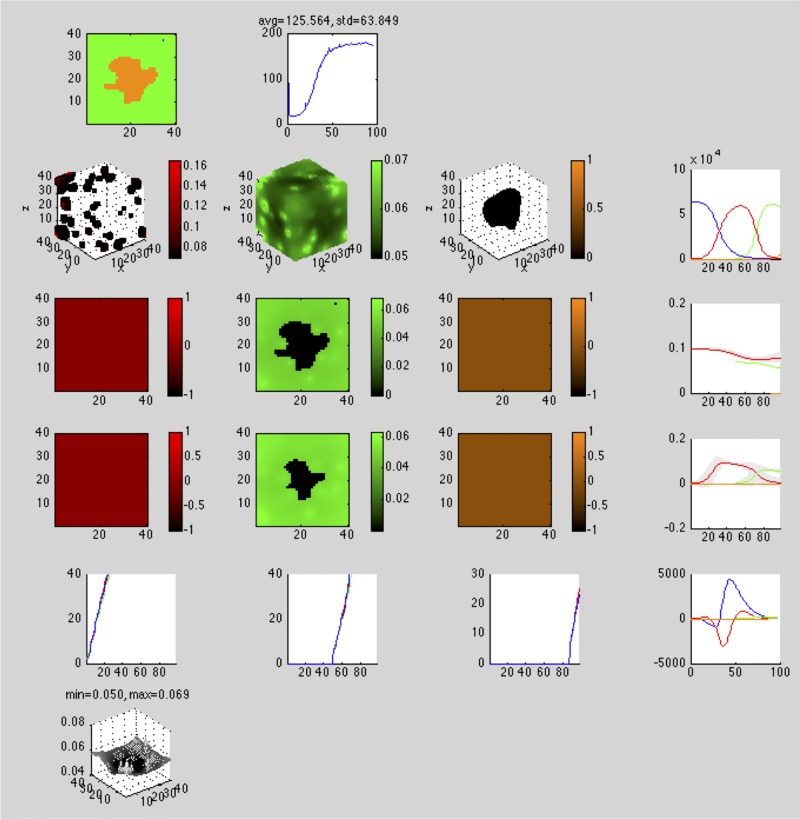
3D simulation console. Simulator console displaying evolving regions of stable chronic hypoxia with many vessels in a 40 × 40 × 40 lattice. The simulation consists of five cell types—vessel (white), *empty* (blue), *viable* (red), *hypoxic* (green), and *necrotic* (orange)—and one particle type, *O*_2_. In row 1, column 1, the cell population at this point in the simulation consists of *hypoxic* and *necrotic* cells. In rows 2-5, columns 1-3, all cell types except vessel and *empty* have fitness and other spatial statistics reported. In row 6, the concentration of *O*_2_ is reported. In column 4, rows 2-5, aggregate cell type statistics are reported in time series. Components of the console displaying 2D plots show the plane *z* = 20.

Figs 1 and 2 show these as they appear in the simulator console for 2D and 3D simulations, respectively.

### Code availability

The simulator was coded in Matlab. It depends on three libraries: Colormap and Colorbar Utilities (to stabilize colormaps in multi-plot figures), freezeColors/unfreezeColors (to stabilize colorbars in multi-plot figures), and Geometric Measures in 2D/3D Images (for computing Minkowski functionals and the Euler-Poincaré characteristic—see Future Work section below).

The simulator code is available in the GitHub repository: https://github.com/aesundstrom/tumor-hypoxia-simulation

### Histology image availability

Our histology images of chronic tumor hypoxia are available in the Harvard Dataverse: http://dx.doi.org/10.7910/DVN/SI32FV

## Results and Discussion

### Metabolic symbiosis (2D)

#### Setup

**Fig 3:**
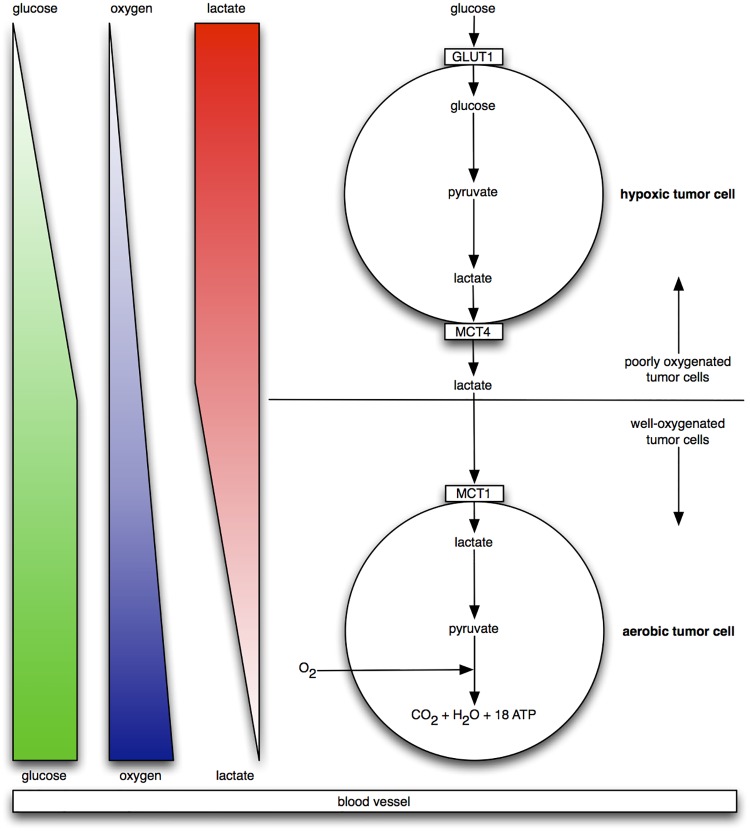
Metabolic symbiosis. A schematic view of the “metabolic symbiosis” [52, 53] between *hypoxic* and *aerobic* tumor cells, where lactate produced by *hypoxic* cells is taken up by *aerobic* cells, which use it as their principal substrate for oxidative phosphorylation. The two cell types thereby mutually regulate their access to energy metabolites. Note the orientation of glucose, oxygen, and lactate gradients with respect to the blood vessel at the bottom.

In this simulation, we have *hypoxic* and *aerobic* tumor cells in the “metabolic symbiosis” scenario illustrated in Fig 3. We have glucose (*glu*), oxygen (*O*_2_), and lactate (*lac*) particles. *Hypoxic* cells consume *glu* at a certain rate, and release *lac* at the same rate. *Aerobic* cells consume *O*_2_ and *lac* at the same rate, and release no particles. In terms of particles and fitness, *glu* positively impacts the fitness of *hypoxic* cells; and *O*_2_ and *lac* positively impact the fitness of *aerobic* cells. All rates and impacts are the same quantities for the two cell types. The one vessel that extends along the bottom row consumes *lac* and releases *glu* and *O*_2_. Both cell types are replaceable and reproductive. The specific quantities mentioned here are given in the tables below.

#### Configuration parameters

We populate configuration parameters as stated in Tables 1–9.

**Table 1:** Diffusion rate of each particle type.

*glu*	*O*_2_	*lac*
1	1	1

**Table 2:** Initial concentration of each particle type.

*glu*	*O*_2_	*lac*
0	0	0.01

**Table 3:** Basal lower-bound concentration of each particle type by cell type.

*cell type*	*glu*	*O*_2_	*lac*
*vessel*	0.1	0.1	0
*empty*	0.1	0.1	0
*hypoxic*	0.1	0.1	0
*aerobic*	0.1	0.1	0

**Table 4:** Basal upper-bound concentration of each particle type by cell type.

*cell type*	*glu*	*O*_2_	*lac*
*vessel*	∞	∞	∞
*empty*	∞	∞	∞
*hypoxic*	∞	∞	∞
*aerobic*	∞	∞	∞

**Table 5:** Consumption rate of each particle type by cell type.

*cell type*	*glu*	*O*_2_	*lac*
*vessel*	0	0	10.0
*empty*	0	0	0
*hypoxic*	1.0	0	0
*aerobic*	0	1.0	1.0

**Table 6:** Release rate for of particle type by cell type.

*cell type*	*glu*	*O*_2_	*lac*
*vessel*	10.0	10.0	0
*empty*	0	0	0
*hypoxic*	0	0	1.0
*aerobic*	0	0	0

**Table 7:** Impact factor of each particle type upon each cell type.

*cell type*	*glu*	*O*_2_	*lac*
*vessel*	0	0	0
*empty*	0	0	0
*hypoxic*	1	0	0
*aerobic*	0	1	1

**Table 8:** Replaceable predicate of each cell type.

*cell type*	*replaceable?*
*vessel*	No
*empty*	Yes
*hypoxic*	Yes
*aerobic*	Yes

**Table 9:** Reproductive predicate of each cell type.

*cell type*	*reproductive?*
*vessel*	No
*empty*	Yes
*hypoxic*	Yes
*aerobic*	Yes

#### Initial conditions

The simulation opens with the initial concentrations of *glu* and *O*_2_ set to zero, and *lac* set to a negligible amount—otherwise, if it too were set to zero, then given the nature of how our simulation computes particle concentrations after cellular consumption and release, it would remain zero throughout the simulation—as specified in Table 2, and lower-bounded basal concentrations of *glu* and *O*_2_, as specified in Table 3. These diffuse with rates specified in Table 1. We initialize the 2D lattice with the top half *hypoxic* cells, the bottom half *aerobic* cells, and place one vessel that extends along the bottom row.

#### Discussion

**Fig 4:**
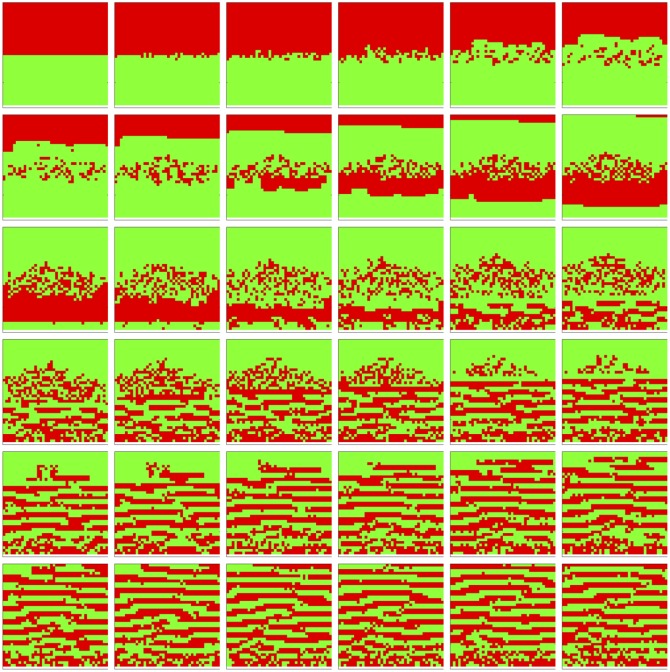
Spatial cell populations during a metabolic symbiosis simulation. Left-to-right, top-to-bottom: 60 generations shown in 36 frames (t = 1, 3, 5, 7, 9, 11, 13, 15, 17, 19, 21, 23, 25, 27, 29, 30, 32, 33, 35, 36, 38, 39, 41, 42, 44, 45, 47, 48, 50, 51, 53, 54, 56, 57, 59, 60). Key: vessel (white), *empty* (blue), *hypoxic* (red), *aerobic* (green) cells.

**Fig 5:**
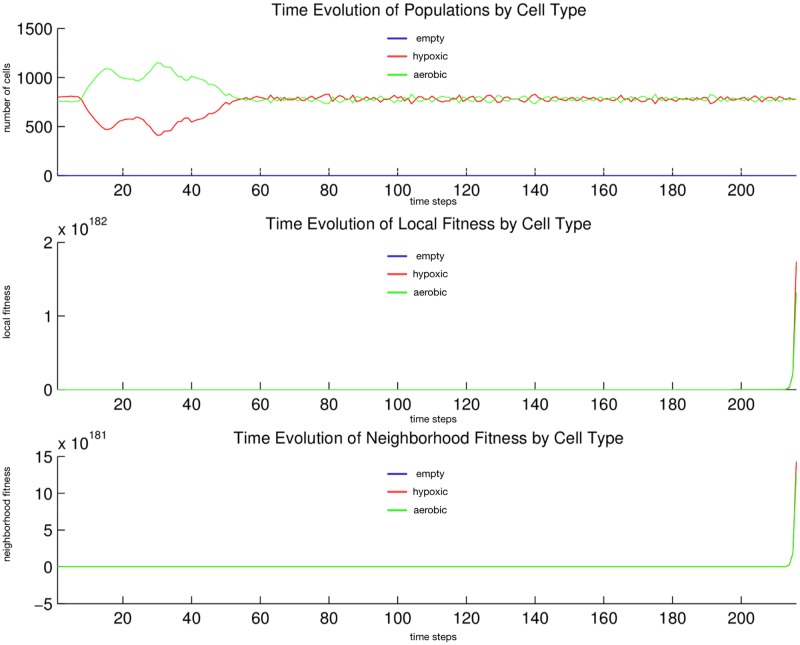
Quantitative cell populations and fitness during a metabolic symbiosis simulation. Time evolution of: populations by cell type (top), local fitness by cell type (middle), and neighborhood fitness by cell type (bottom). In the top, the horizontal axis denotes simulation time steps, and the vertical axis denotes number of cells; in the middle and bottom, the horizontal axes denote simulation time steps, and the vertical axes denote dimensionless fitness scores. In the middle and bottom, mean curves are plotted and gray regions above and below show the respective standard deviations. Key: *empty* (blue), *hypoxic* (red), *aerobic* (green) cells.

We observe in Fig 4 that the *hypoxic* and *aerobic* cell populations immediately begin to mix at their horizontal interface. Then a thick horizontal layer of *aerobic* cells pulls away and migrates upward; transiently, it appears that the populations of *hypoxic* and *aerobic* cells have swapped positions. Then a thick horizontal layer of *aerobic* cells pulls away and migrates downward. This inversion and pull-away migration repeats at successively smaller length scales, all the while maintaining its near perfect horizontality, until the entire area is covered in a regular striation pattern: horizontal, equal thickness layers of *aerobic* and *hypoxic* cells alternate from top to bottom. This pattern is stable for the duration of the simulation, and perhaps indefinitely. In Fig 5 we observe that since the two cell populations always fill the entire area, their population dynamics is always zero-sum in character. From generation 10 to 50, *aerobic* cells dominate; but from generation 50 onward, the two populations converge at, and oscillate about, the same mean size. We expect neither the reaction-diffusion [56] type emergent striation pattern nor the population size rebalancing.

That being said, we struggle to understand our results in a biologically meaningful way. While cell self-sorting is possible in principle, we are unaware of any *in vitro* or *in vivo* studies of metabolic symbiosis that observe alternating striation patterns of *hypoxic* and *aerobic* cell types. Though we base our simulation of metabolic symbiosis on an illustrative explanation in Sonveaux, *et al* [53], perhaps our assumption that *aerobic* cells are totally dependent on lactate and do not consume glucose is unrealistic. The striations may also result from numerical artifacts emerging from the fitness calculations and initial conditions; for instance, this could arise from resonance created by the kinetics of the system and the simulation time step. In the simulation, all cells move zero or one lattice point in a given time step, so the discrete nature of the cell replacement may result in an artifact; in a true biological context, this would be smoothed by varying migration rates in the locality of any given cell. In a follow-up study, we will try to reproduce these results over a range of particle diffusivities and cell migration rates—since we use fitness-based probabilistic replacement as a surrogate for modeling explicit cell migration, we would explore a range of fitness parameters for this purpose.

We ran this simulation 10 times and observed the results were similar. First, we analyzed the spatial frequency of the striations over the simulations. Each simulation produces a spatial cell occupation map as it evolves and as its final output. To show the consistent spatial periodicity of the striations of *aerobic* and *hypoxic* cells across simulations, we used Matlab’s FFT2 function to transform each resulting occupation map into the frequency domain, then examined the mean FFT2 magnitude over 10 simulations. See S1 Fig. Notice the two energy loci above and below the center. They are distant from the center due to the sharp boundaries (and thus high spatial frequency information) of the striations. They are vertically oriented from the center because the striations tend to be horizontal and are thus perpendicular to the FFT2 magnitude orientation. And the loci are tightly clustered, indicating the consistent periodicity of striations between simulations. S2 and S3 Figs attest to the dispersion and noise in the spatial frequency data. The maximum standard deviation is 0.43 times the maximum mean value, and no regions of high noise-to-signal ratio colocate with the two energy loci; rather, the noise appears uniformly distributed across the energy surface. Looking at the population evolution (S4–S6 Figs), the overlaid simulation trajectories show the similar population dynamics. In S5 Fig, notice the standard deviations are identical for *hypoxic* (red) and *aerobic* (green) populations—green is overlaid atop red—due to their zero-sum relationship; a gain in one population is precisely the loss in the other, and vice-versa. The maximum standard deviation is 0.12 times the maximum mean value. In S6 Fig, unlike their respective standard deviations, the populations have differing coefficients of variation since their respective denominators (mean population sizes) differ. The maximum coefficient of variation is 0.12.

### Tumor-stroma signaling (2D)

#### Setup

**Fig 6:**
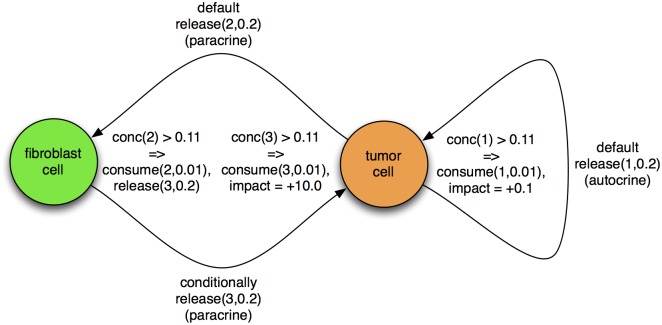
Tumor-stroma signaling. A schematic view of the tumor-stroma signaling described below, and quantified in Tables 10–19.

In this simulation, we have *epithelial*, *fibroblast*, *tumor*, and *inert* cells. Three particle types play roles in the following scenario. In a middle of a row of *epithelial* cells, one of them transforms into a *tumor* cell. The *epithelial* cells are separated from the *fibroblast* cells as these are embedded in a thick layer of (*inert*) extracellular matrix. Proliferating (*tumor*) cells (autocrine) signal themselves with particle type 1. When this reaches a sufficient concentration, affected *tumor* cells begin consuming it and their fitness increases. Simultaneously, *tumor* cells (paracrine) signal nearby *fibroblast* cells residing in the (*inert*) extracellular matrix with particle type 2. When this reaches a sufficient concentration, affected *fibroblast* cells begin consuming it and immediately releasing particle type 3. When this reaches a sufficient concentration, affected *tumor* cells begin consuming it and their fitness increases by a substantial factor, above and beyond that from its autocrine signaling. In this way, our conditional logic implements a simple signaling and adaptation system between *tumor* and *fibroblast* cells, illustrated schematically in Fig 6. By default, no cells consume particles, and only *tumor* cells release particle types 1 and 2 at the same rate, to kick off autocrine and paracrine signaling, respectively. By default, no impact factors are defined; they are only conditionally applied. Only *empty* cells are replaceable, and only *tumor* cells are reproductive; the others are effectively inert. The specific quantities mentioned here are given in the tables below.

#### Configuration parameters

We populate configuration parameters as stated in Tables 10–19.

**Table 10:** Diffusion rate of each particle type.

*pt 1*	*pt 2*	*pt 3*
10	0.1	0.1

**Table 11:** Initial concentration of each particle type.

*pt 1*	*pt 2*	*pt 3*
0.1	0.1	0.1

**Table 12:** Basal lower-bound concentration of each particle type by cell type.

*cell type*	*pt 1*	*pt 2*	*pt 3*
*vessel*	0	0	0
*empty*	0	0	0
*epithelial*	0	0	0
*fibroblast*	0	0	0
*tumor*	0	0	0
*inert*	0	0	0

**Table 13:** Basal upper-bound concentration of each particle type by cell type.

*cell type*	*pt 1*	*pt 2*	*pt 3*
*vessel*	∞	∞	∞
*empty*	∞	∞	∞
*epithelial*	∞	∞	∞
*fibroblast*	∞	∞	∞
*tumor*	∞	∞	∞
*inert*	∞	∞	∞

**Table 14:** Consumption rate of each particle type by cell type.

*cell type*	*pt 1*	*pt 2*	*pt 3*
*vessel*	0	0	0
*empty*	0	0	0
*epithelial*	0	0	0
*fibroblast*	0	0	0
*tumor*	0	0	0
*inert*	0	0	0

**Table 15:** Release rate for of particle type by cell type.

*cell type*	*pt 1*	*pt 2*	*pt 3*
*vessel*	0	0	0
*empty*	0	0	0
*epithelial*	0	0	0
*fibroblast*	0	0	0
*tumor*	0.2	0.2	0
*inert*	0	0	0

**Table 16:** Impact factor of each particle type upon each cell type.

*cell type*	*pt 1*	*pt 2*	*pt 3*
*vessel*	0	0	0
*empty*	0	0	0
*epithelial*	0	0	0
*fibroblast*	0	0	0
*tumor*	0	0	0
*inert*	0	0	0

**Table 17:** Replaceable predicate of each cell type.

*cell type*	*replaceable?*
*vessel*	No
*empty*	Yes
*epithelial*	No
*fibroblast*	No
*tumor*	No
*inert*	No

**Table 18:** Reproductive predicate of each cell type.

*cell type*	*reproductive?*
*vessel*	No
*empty*	Yes
*epithelial*	No
*fibroblast*	No
*tumor*	Yes
*inert*	No

**Table 19:** Conditional triggers and actions for those cell types so configured.

*cell type*	*triggers*	*actions*
*fibroblast*	*ρ*_2_ > 0.11	*c*_*fibroblast*,2_ ← 0.01, *r*_*fibroblast*,3_ ← 0.2
*tumor*	*ρ*_1_ > 0.11	*c*_*tumor*,1_ ← 0.01, *σ*_*tumor*,1_ ← 0.1
*tumor*	*ρ*_3_ > 0.11	*c*_*tumor*,3_ ← 0.01, *σ*_*tumor*,3_ ← 10

#### Initial conditions

The simulation opens with the initial concentrations of particle types 1, 2, and 3 set to the same value, as specified in Table 11, with no lower- or upper-bounded basal concentrations set. These diffuse with rates specified in Table 10. We initialize the 2D lattice with a horizontal monolayer of *epithelial* cells, located about two-thirds down from the top, in the middle of which we place a single *tumor* cell. Below this, we lay down a thick layer of sub-*epithelial* (*inert*) extracellular matrix. Within this layer, we place a random scattering of *fibroblast* cells such that their density increases nonlinearly in a rightward direction—this amounts in most of the *fibroblast* cells populating the rightmost third of the area.

#### Discussion

**Fig 7:**
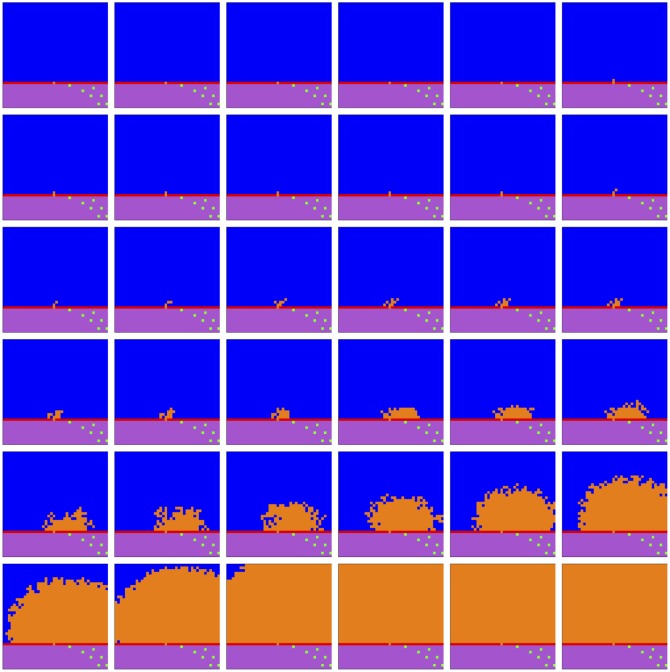
Spatial cell populations during a tumor-stroma signaling simulation. Time evolution of cell populations. Left-to-right, top-to-bottom: 260 generations shown in 36 frames (t = 1, 8, 15, 22, 29, 36, 43, 50, 58, 65, 73, 80, 88, 95, 103, 110, 118, 125, 133, 140, 148, 155, 163, 170, 178, 185, 193, 200, 208, 215, 223, 230, 238, 245, 253, 260). Key: *vessel* (white), *empty* (blue), *epithelial* (red), *fibroblast* (green), *tumor* (orange), *inert* (purple).

**Fig 8:**
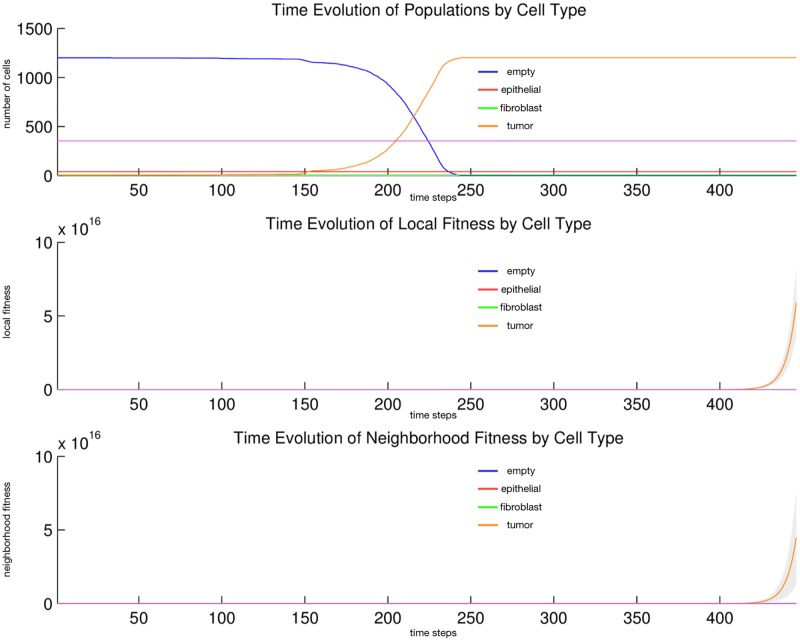
Quantitative cell populations and fitness during a tumor-stroma signaling simulation. Time evolution of: populations by cell type (top), local fitness by cell type (middle), and neighborhood fitness by cell type (bottom). In the top, the horizontal axis denotes simulation time steps, and the vertical axis denotes number of cells; in the middle and bottom, the horizontal axes denote simulation time steps, and the vertical axes denote dimensionless fitness scores. In the middle and bottom, mean curves are plotted and gray regions above and below show the respective standard deviations. Key: *empty* (blue), *epithelial* (red), *fibroblast* (green), *tumor* (orange), *inert* (purple).

We observe in Fig 7 that *tumor* cell proliferation is very slow at first, as the autocrine signaling causes only a small increase in affected *tumor* cell fitness. It is not until much later, when *fibroblast* paracrine signaling reaches the *tumor* cells that their proliferation becomes noticeable, due to the consequent much higher fitness impact on the affected *tumor* cells, and they eventually take over the replaceable area. Notice that this faster growth moves in a decidedly rightward direction, toward the source of the signaling gradient, the rightwardly dense *fibroblast* cells. After a fashion, radial outward growth dominates and the *tumor* contour becomes circular. In Fig 8 we observe linear growth in the *tumor* population size from generation 145 to 155, then exponential growth from generation 155 to 250, where the population size saturates at the full replaceable area.

We ran this simulation 10 times and observed the results were similar. See S7 Fig. Notice the onset of *tumor* cell population growth varies by 120 time units (due to the random positioning of reciprocally-signaling *fibroblast* cells, and thus the variability in the onset of the positive growth feedback), but once growth onset occurs, the shape and slope of that growth is similar. S8 and S9 Figs attest to the respective dispersion and noise in the population dynamics. The apparently large standard deviation and coefficient of variation values are due to the variation in growth onset times, as can be seen in the simulation trajectories, and trying to fit them to a unimodal Gaussian distribution.

### Stable local chronic hypoxia with many vessels (2D)

#### Setup

In this simulation, we have *viable*, *hypoxic*, and *necrotic* tumor cells that correspond to those we see in our anti-pimonidazole stain images. The *viable* and *hypoxic* cells consume *O*_2_ at a certain rate, and release no particles. The conditional logic implements a simple state machine in the following way. Wherever *O*_2_ falls below a threshold, *viable* cells become (“jump” to) *hypoxic* cells—identical in every way except as follows. Wherever *O*_2_ rises above that threshold, *hypoxic* cells become *viable* again; and wherever *O*_2_ falls below an even lower threshold, *hypoxic* cells become *necrotic* cells. Once a cell becomes *necrotic* it has entered an absorbing state and its behavior is completely inert: it has no consumption or release profile, and it is neither replaceable nor reproductive. The specific quantities mentioned here are given in the tables below.

#### Configuration parameters

We populate configuration parameters as stated in Tables 20–29.

**Table 20:** Diffusion rate of each particle type.

*O*_2_
0.12

**Table 21:** Initial concentration of each particle type.

*O*_2_
0.1

**Table 22:** Basal lower-bound concentration of each particle type by cell type.

*cell type*	*O*_2_
*vessel*	0
*empty*	0
*viable*	0
*hypoxic*	0
*necrotic*	0

**Table 23:** Basal upper-bound concentration of each particle type by cell type.

*cell type*	*O*_2_
*vessel*	∞
*empty*	∞
*viable*	∞
*hypoxic*	∞
*necrotic*	∞

**Table 24:** Consumption rate of each particle type by cell type.

*cell type*	*O*_2_
*vessel*	0
*empty*	0
*viable*	0.01
*hypoxic*	0.01
*necrotic*	0

**Table 25:** Release rate for of particle type by cell type.

*cell type*	*O*_2_
*vessel*	0.2
*empty*	0
*viable*	0
*hypoxic*	0
*necrotic*	0

**Table 26:** Impact factor of each particle type upon each cell type.

*cell type*	*O*_2_
*vessel*	0
*empty*	0
*viable*	1
*hypoxic*	1
*necrotic*	0

**Table 27:** Replaceable predicate of each cell type.

*cell type*	*replaceable?*
*vessel*	No
*empty*	Yes
*viable*	No
*hypoxic*	No
*necrotic*	No

**Table 28:** Reproductive predicate of each cell type.

*cell type*	*reproductive?*
*vessel*	No
*empty*	Yes
*viable*	Yes
*hypoxic*	Yes
*necrotic*	No

**Table 29:** Conditional triggers and actions for those cell types so configured.

*cell type*	*triggers*	*actions*
*viable*	*ρ*_1_ < 0.07	jump to *hypoxic*
*hypoxic*	*ρ*_1_ < 0.05	jump to *necrotic*
*hypoxic*	*ρ*_1_ > 0.07	jump to *viable*

#### Initial conditions

The simulation opens with the initial concentration of *O*_2_ specified in Table 21. This diffuses with a rate specified in Table 20. We initialize the 2D lattice with *empty* cells; we place one *viable* cell in the center, and 10 vessels randomly about. Vessels consume no particles and release *O*_2_ at the rate specified in Table 25.

#### Discussion

**Fig 9:**
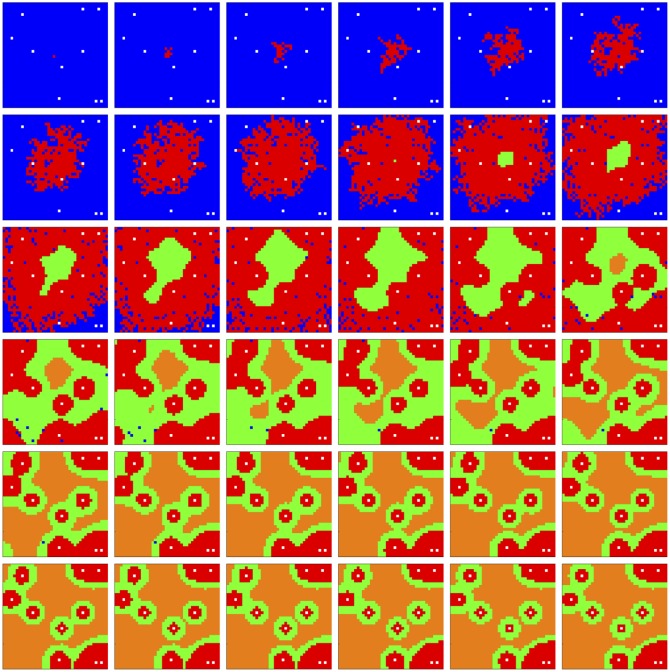
Spatial cell populations during a stable local chronic hypoxia with many vessels (2D) simulation. Time evolution of cell populations. Left-to-right, top-to-bottom: 220 generations shown in 36 frames (t = 1, 7, 13, 19, 25, 31, 37, 43, 49, 55, 61, 67, 73, 79, 85, 91, 97, 103, 110, 116, 123, 129, 136, 142, 149, 155, 162, 168, 175, 181, 188, 194, 201, 207, 214, 220). Key: *vessel* (white), *empty* (blue), *viable* (red), *hypoxic* (green), *necrotic* (orange).

**Fig 10:**
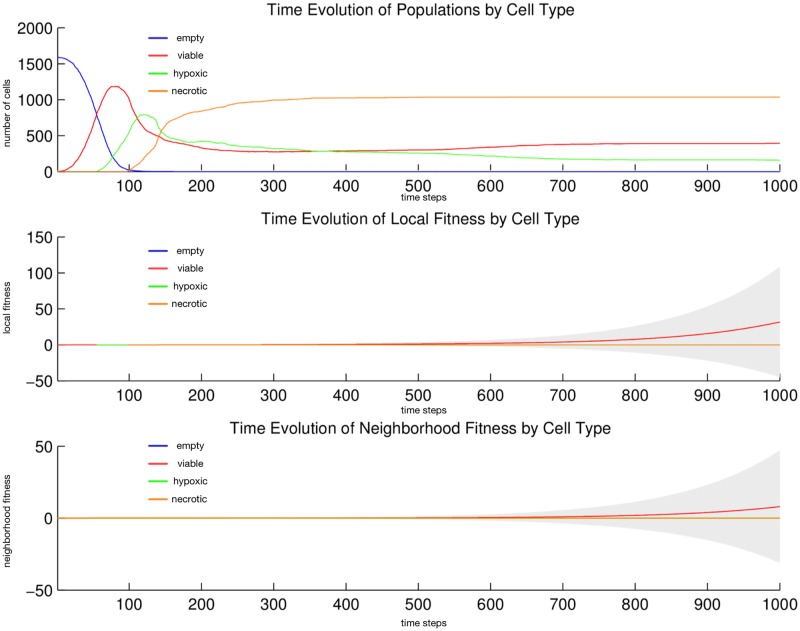
Quantitative cell populations and fitness during a stable local chronic hypoxia with many vessels (2D) simulation. Time evolution of: populations by cell type (top), local fitness by cell type (middle), and neighborhood fitness by cell type (bottom). In the top, the horizontal axis denotes simulation time steps, and the vertical axis denotes number of cells; in the middle and bottom, the horizontal axes denote simulation time steps, and the vertical axes denote dimensionless fitness scores. In the middle and bottom, mean curves are plotted and gray regions above and below show the respective standard deviations. Key: *empty* (blue), *viable* (red), *hypoxic* (green), *necrotic* (orange).

We observe the following sequence of events in Fig 9. *Viable* cells proliferate radially outward from the center. Once most of the area is covered with *viable* cells, then *hypoxic* cells appear in the center. *Hypoxic* cells grow radially outward at a similar rate to the *viable* cells just beyond them. They approach vessels (surrounded by *viable* cells) up to some radial distance away from each vessel, the zone into which *O*_2_ diffuses with a sufficient concentration to maintain *viable* cells. Once about half of the area is covered with *hypoxic* cells, then *necroic* cells appear in the center. *Necrotic* cells grow radially outward at a similar rate to the *hypoxic* and *viable* cells just beyond them. They approach vessels (surrounded by concentrically situated *viable* and *hypoxic* cells) up to some radial distance away from each vessel, the zone into which *O*_2_ diffuses with a sufficient concentration to maintain *viable* and *hypoxic* cells. We eventually observe islands of concentrically situated *viable* and *hypoxic* cells, surrounded by a sea of *necrotic* cells, similar to what we see in our anti-pimonidazole stain images. This arrangement is stable for awhile, before the imbalance of three factors related to *O*_2_—diffusion rate (Table 20), vessel release rate (Table 25), and *viable* and *hypoxic* consumption rate (Table 24)—allow *O*_2_ concentration to climb out of control, at least in certain locales where random vessels may be close together, and eventually convert all of the *hypoxic* cells back into *viable* cells. We observe the succession of *viable* → *hypoxic* → *necrotic* cell populations in Fig 10. The initial decay of *viable* and *hypoxic* cell populations is due to their proliferating outside of the spatial dimensions of the simulation while simultaneously being replaced by the succeeding population. Since *necrotic* cells cannot be replaced, their population size monotonically increases. Beginning at generation 200 and continuing to the end of the simulation, the growth in the *viable* cell population at the expense of the *hypoxic* cell population occurs for the reasons just discussed.

We ran this simulation 10 times and observed the results were similar, modulo some pattern difference in the bands of *viable* and *hypoxic* cells, and in the growth and decay in those populations, as would be expected given the random placement of vessels with each simulation run. See S10–S12 Figs. In S11 Fig, the apparently large and growing standard deviation values after time 150 is due to the randomly placed vessels causing differing patterns of growth and decay in the *viable* and *hypoxic* populations. In S12 Fig, despite the standard deviation values after time 150, we see the corresponding CV values drop sharply and remain low. In each 2D simulation, 10 vessels were randomly placed in the simulation area, so across simulations we had 10 × 10 = 100 candidate vessels to measure. Of these, 6 vessels were far enough from the edges and far enough from each other to provide undistorted measurement of the area and radius of *viable* and *hypoxic* cells about the vessel. See S1 Table, rows 1-4. Here the statistics describe a band of *viable* cells having mean thickness 1.60 about the vessel, and a band of *hypoxic* cells having mean thickness of 3.87 − 1.60 = 2.27 around the *viable* ones. These low variance measures closely match our observation of simulation output and support the structural similarity of band formation across respective simulations.

### Stable local chronic hypoxia with many vessels (3D)

#### Setup

In this simulation, we have *viable*, *hypoxic*, and *necrotic* tumor cells that correspond to those we see in our anti-pimonidazole stain images. The *viable* and *hypoxic* cells consume *O*_2_ at a certain rate, and release no particles. The conditional logic implements a simple state machine in the following way. Wherever *O*_2_ falls below a threshold, *viable* cells become (“jump” to) *hypoxic* cells—identical in every way except as follows. Wherever *O*_2_ rises above that threshold, *hypoxic* cells become *viable* again; and wherever *O*_2_ falls below an even lower threshold, *hypoxic* cells become *necrotic* cells. Once a cell becomes *necrotic* it has entered an absorbing state and its behavior is completely inert: it has no consumption or release profile, and it is neither replaceable nor reproductive. The specific quantities mentioned here are given in the tables below.

#### Configuration parameters

We populate configuration parameters as stated in Tables 30–39.

**Table 30:** Diffusion rate of each particle type.

*O*_2_
0.12

**Table 31:** Initial concentration of each particle type.

*O*_2_
0.1

**Table 32:** Basal lower-bound concentration of each particle type by cell type.

*cell type*	*O*_2_
*vessel*	0
*empty*	0
*viable*	0
*hypoxic*	0
*necrotic*	0

**Table 33:** Basal upper-bound concentration of each particle type by cell type.

*cell type*	*O*_2_
*vessel*	∞
*empty*	∞
*viable*	∞
*hypoxic*	∞
*necrotic*	∞

**Table 34:** Consumption rate of each particle type by cell type.

*cell type*	*O*_2_
*vessel*	0
*empty*	0
*viable*	0.01
*hypoxic*	0.01
*necrotic*	0

**Table 35:** Release rate for of particle type by cell type.

*cell type*	*O*_2_
*vessel*	0.2
*empty*	0
*viable*	0
*hypoxic*	0
*necrotic*	0

**Table 36:** Impact factor of each particle type upon each cell type.

*cell type*	*O*_2_
*vessel*	0
*empty*	0
*viable*	1
*hypoxic*	1
*necrotic*	0

**Table 37:** Replaceable predicate of each cell type.

*cell type*	*replaceable?*
*vessel*	No
*empty*	Yes
*viable*	No
*hypoxic*	No
*necrotic*	No

**Table 38:** Reproductive predicate of each cell type.

*cell type*	*reproductive?*
*vessel*	No
*empty*	Yes
*viable*	Yes
*hypoxic*	Yes
*necrotic*	No

**Table 39:** Conditional triggers and actions for those cell types so configured.

*cell type*	*triggers*	*actions*
*viable*	*ρ*_1_ < 0.07	jump to *hypoxic*
*hypoxic*	*ρ*_1_ < 0.05	jump to *necrotic*
*hypoxic*	*ρ*_1_ > 0.07	jump to *viable*

#### Initial conditions

The simulation opens with the initial concentration of *O*_2_ specified in Table 31. This diffuses with a rate specified in Table 30. We initialize the 3D lattice with *empty* cells; we place one *viable* cell in the center, and 100 vessels randomly about. Vessels consume no particles and release *O*_2_ at the rate specified in Table 35.

#### Discussion

**Fig 11:**
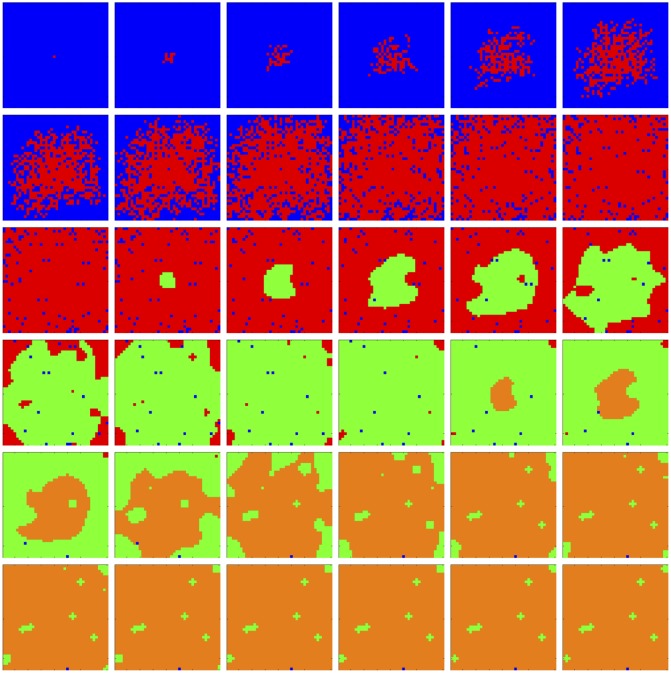
Spatial cell populations during a stable local chronic hypoxia with many vessels (3D) simulation. Time evolution of cell populations on the plane *z* = 20. Left-to-right, top-to-bottom: 150 generations shown in 36 frames (t = 1, 5, 9, 13, 17, 21, 25, 29, 33, 37, 41, 45, 49, 53, 57, 61, 65, 69, 74, 78, 83, 87, 92, 96, 101, 105, 110, 114, 119, 123, 128, 132, 137, 141, 146, 150). Key: *vessel* (white), *empty* (blue), *viable* (red), *hypoxic* (green), *necrotic* (orange).

**Fig 12:**
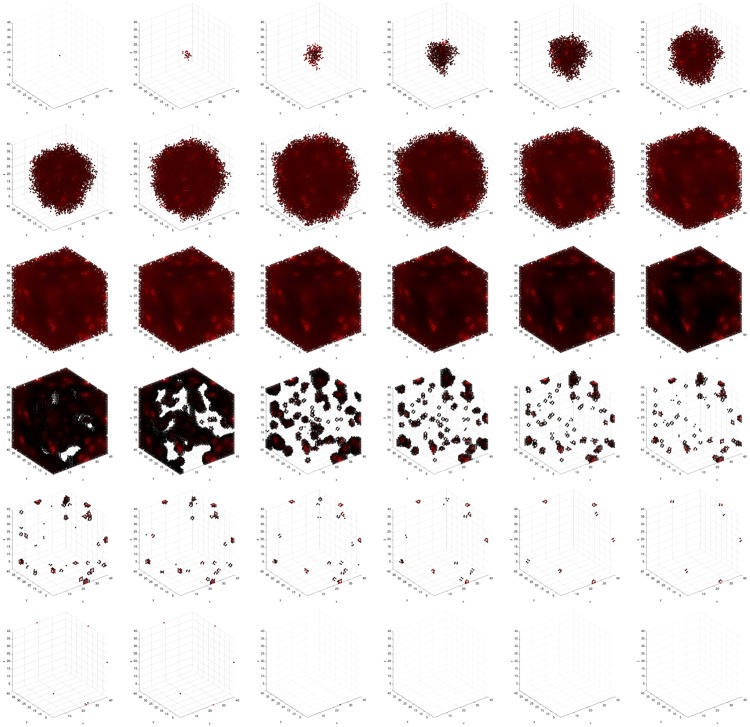
Spatial time evolution of local fitness for *viable* (red) cells during a stable local chronic hypoxia with many vessels (3D) simulation. Left-to-right, top-to-bottom: 150 generations shown in 36 frames (t = 1, 5, 9, 13, 17, 21, 25, 29, 33, 37, 41, 45, 49, 53, 57, 61, 65, 69, 74, 78, 83, 87, 92, 96, 101, 105, 110, 114, 119, 123, 128, 132, 137, 141, 146, 150).

**Fig 13:**
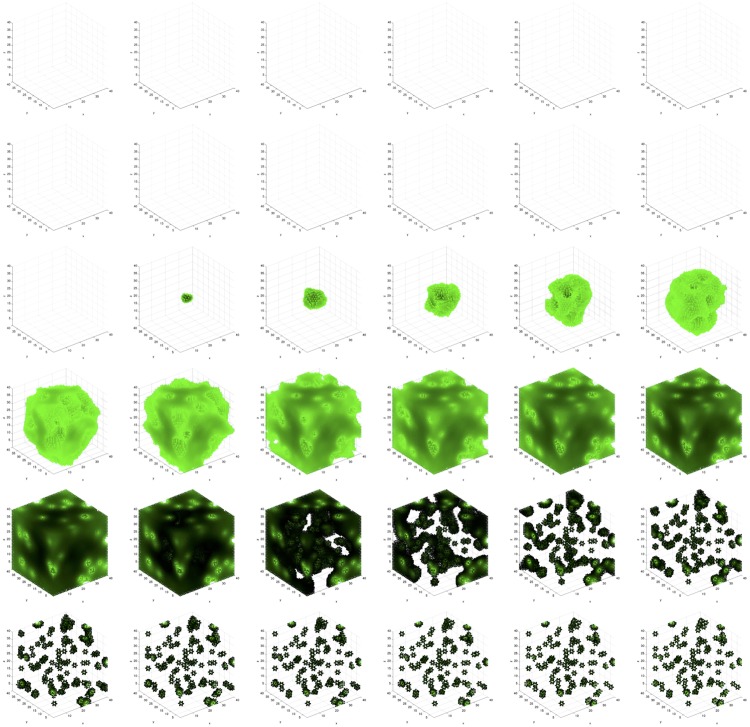
Spatial time evolution of local fitness for *hypoxic* (green) cells during a stable local chronic hypoxia with many vessels (3D) simulation. Left-to-right, top-to-bottom: 150 generations shown in 36 frames (t = 1, 5, 9, 13, 17, 21, 25, 29, 33, 37, 41, 45, 49, 53, 57, 61, 65, 69, 74, 78, 83, 87, 92, 96, 101, 105, 110, 114, 119, 123, 128, 132, 137, 141, 146, 150).

**Fig 14:**
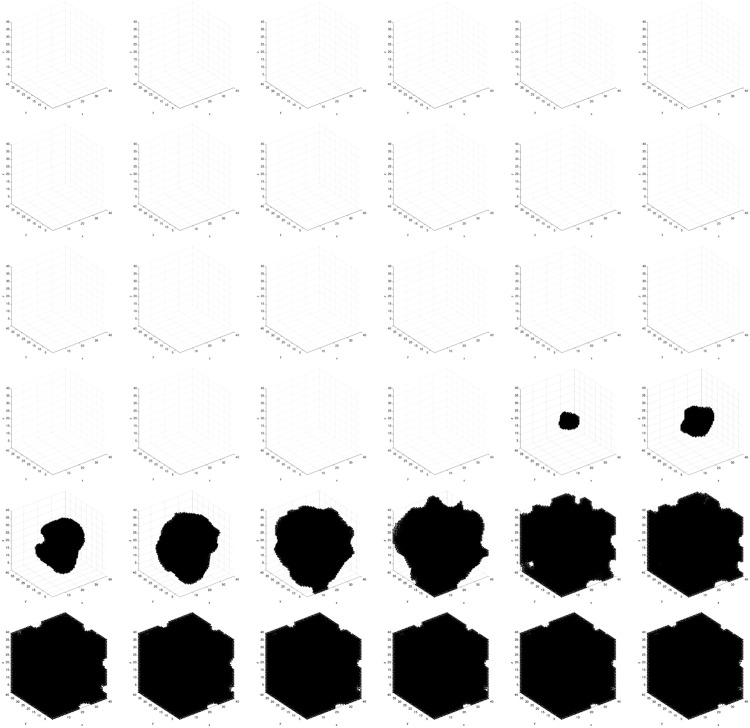
Spatial time evolution of local fitness for *necrotic* (orange) cells during a stable local chronic hypoxia with many vessels (3D) simulation. Left-to-right, top-to-bottom: 150 generations shown in 36 frames (t = 1, 5, 9, 13, 17, 21, 25, 29, 33, 37, 41, 45, 49, 53, 57, 61, 65, 69, 74, 78, 83, 87, 92, 96, 101, 105, 110, 114, 119, 123, 128, 132, 137, 141, 146, 150).

**Fig 15:**
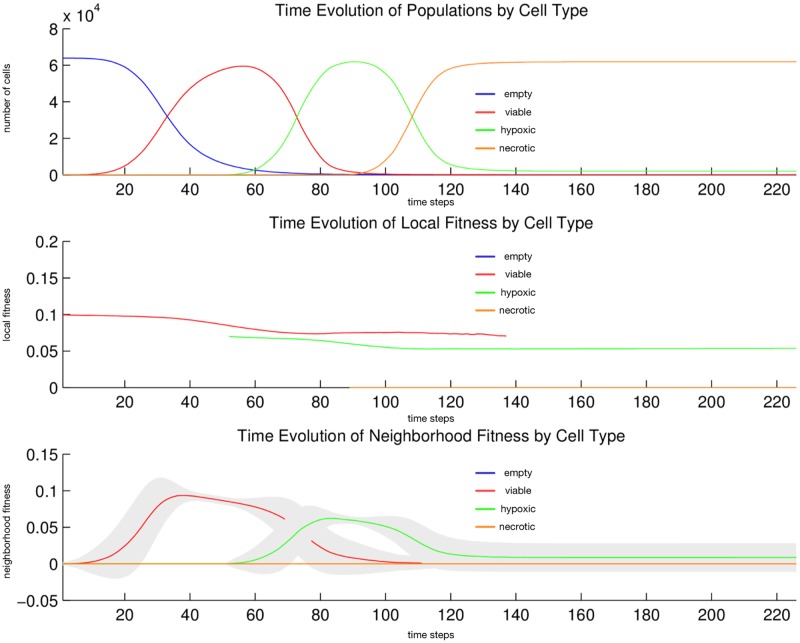
Quantitative cell populations and fitness during a stable local chronic hypoxia with many vessels (3D) simulation. Time evolution of: populations by cell type (top), local fitness by cell type (middle), and neighborhood fitness by cell type (bottom). In the top, the horizontal axis denotes simulation time steps, and the vertical axis denotes number of cells; in the middle and bottom, the horizontal axes denote simulation time steps, and the vertical axes denote dimensionless fitness scores. In the middle and bottom, mean curves are plotted and gray regions above and below show the respective standard deviations. Key: *empty* (blue), *viable* (red), *hypoxic* (green), *necrotic* (orange).

We observe the following sequence of events in Figs 11–14. *Viable* cells proliferate radially outward from the center. Once most of the volume is covered with *viable* cells, then *hypoxic* cells appear in the center. *Hypoxic* cells grow radially outward at a similar rate to the *viable* cells just beyond them. They approach vessels (surrounded by *viable* cells) up to some radial distance away from each vessel, the zone into which *O*_2_ diffuses with a sufficient concentration to maintain *viable* cells. Once about half of the volume is covered with *hypoxic* cells, then *necrotic* cells appear in the center. *Necrotic* cells grow radially outward at a similar rate to the *hypoxic* and *viable* cells just beyond them. They approach vessels (surrounded by concentrically situated *viable* and *hypoxic* cells) up to some radial distance away from each vessel, the zone into which *O*_2_ diffuses with a sufficient concentration to maintain *viable* and *hypoxic* cells. We eventually observe islands of concentrically situated *viable* and *hypoxic* cells, surrounded by a sea of *necrotic* cells, similar to what we see in our anti-pimonidazole stain images. This arrangement is stable for awhile, before the remaining *viable* cells become *hypoxic* cells. These *hypoxic* cell islands persist indefinitely. We observe the succession of *viable* → *hypoxic* → *necrotic* cell populations in Fig 15. The initial decay of *viable* and *hypoxic* cell populations is due to their proliferating outside of the spatial dimensions of the simulation while simultaneously being replaced by the succeeding population. Since *necrotic* cells cannot be replaced, their population size monotonically increases. By generation 125, *necrotic* cells have taken over the volume, except for the islands of *hypoxic* cells.

We ran this simulation 10 times and observed the results were similar, modulo some pattern difference in the shells of *viable* and *hypoxic* cells, and in the growth and decay in those populations, as would be expected given the random placement of vessels with each simulation run. See S13–S15 Figs. In S14 Fig, after the successive fluctuations in *viable* then *hypoxic* then *necrotic* populations (after time 150), standard deviation values drop sharply, as we expect from stable co-existing populations at nearly identical sizes across simulations. In S15 Fig, after the successive fluctuations in *viable* then *hypoxic* then *necrotic* populations (after time 150), coefficient of variation values drop sharply, as we expect from stable co-existing populations at nearly identical sizes across simulations. The higher CV for the *viable* population size is due to the denominator (mean population size) fluctuating near zero consistently across simulations. In each 3D simulation, 100 vessels were randomly placed in the simulation volume, so across simulations we had 10 × 100 = 1000 candidate vessels to measure. Of these, 16 vessels were far enough from the edges and far enough from each other to provide undistorted measurement of the volume and radius of *viable* and *hypoxic* cells about the vessel. See S1 Table, rows 5-8. Here the statistics describe a shell of *viable* cells having mean thickness 0.00 (absent) about the vessel, and a shell of *hypoxic* cells having mean thickness of 1.43 − 0.00 = 1.43 around the *viable* ones; the absence of *viable* cells and thinner accumulation of *hypoxic* cells in the 3D case is most likely due to greater diffusion of *O*_2_. These low variance measures closely match our observation of simulation output and support the structural similarity of shell formation across respective simulations.

## Conclusion

### Our contributions

We developed a spatially-resolved, mixed-population simulation which is minimal, fast, extensible, and adaptable. First, we can support any number of cell types and any number of particle types (each with its own diffusion rate). Second, each cell type has default behaviors, as before, and conditional behaviors, which can implement phenotypical adaptations and mutations, and state machines composed of two or more cell types. Third, initial, and upper- and lower-bounded basal concentrations can be set for each particle type. Fourth, each cell type can be replaceable or not, and reproductive or not. Fifth, initial lattice occupation can be delayed to establish complex diffusion gradients to form prior to simulation.

### Our findings

Regarding the simulation, we found that with a few simple ingredients—space, distinct particle types with their own diffusion rates, distinct cell types with default consumption and release profiles, and conditional logic to implement cell-type-specific local adaptation—we captured a number of interesting features and phenomena. First, in a study of asymmetric fitness with one vessel (not shown here), we observed sustained coexistence of two populations; one was dominant but did not drive the other to extinction. Second, in Results subsection “Metabolic symbiosis (2D)”, we observed emergent spatial self-organization among *hypoxic* and *aerobic* cells into a stable striation pattern (without conditional logic), followed by population size rebalancing. Third, in Results subsection “Tumor-stroma signaling (2D)”, we were able to implement a functioning system of autocrine and reciprocal paracrine signaling. Fourth, in Results subsections “Stable local chronic hypoxia with many vessels (2D)” and “Stable local chronic hypoxia with many vessels (3D)”, we observed emergent 2D and 3D necrotic cores, respectively; this was followed by emergent formation of local regions of spatially and numerically stable *viable-hypoxic* cell populations that were concentrically oriented, in 2D and 3D, respectively, over different vascular densities. In terms of relative orientation, composition, and dimensions, these simulated formations were similar to what we observed in our anti-pimonidazole stain images. This is especially true where the randomized vasculature was more dense, thereby breaking diffusion symmetries and giving rise to more realistic *viable-hypoxic* agglomerations.

Regarding the biology of chronic hypoxia, in Results subsections “Stable local chronic hypoxia with many vessels (2D)” and “Stable local chronic hypoxia with many vessels (3D)”, we found there was an instability in the *viable-hypoxic* cell population balance. Either over-oxygenation eventually converted all *hypoxic* cells to *viable* cells, or under-oxygenation did the reverse and then eventually these *hypoxic* cells became *necrotic* cells. We observed the oxygenation balance derived from three rates related to *O*_2_: vessel release rate, the local population’s average consumption rate, and the diffusion rate. There are at least two ways to explain this: (1) histology data show a delicate balance that is stable, and therefore in evidence everywhere; or (2) the balance is a transient phenomenon, and our histology happens to have caught one early stage. But which is it? In other words, what is the relationship between tumor age and average intra-tumor chronic hypoxia? We know tumor age correlates positively with degree of vasculature, and therefore density of oxygenation, so we can design experiments to address this question. In a follow-up study, we will fully explore the three rate parameters responsible for the patterns of chronic hypoxia in light of these questions.

Whatever the cancer disease context, one could re-simulate the cell populations after adding various therapeutic strategies (e.g., dosage frequency, cocktail therapy, immunotherapy) by way of different delivery modalities (e.g., from explicitly represented vessels, or from a non-specific source of diffusion) to model therapeutic effects and to understand when resistance develops. These would produce additional emergent dynamics that would be worth analyzing.

### Limitations and additional considerations

First, it may turn out that a lattice-based simulation is too limited to capture properties related to cell crowdedness, which is arguably essential for modeling density-derived control of tumor cell population growth, and emergent geometric and spatial organization. It may also have significance for modeling emerging local, stable regions of chronic hypoxia. Without an explicit notion of crowdedness, one cannot correctly model contact inhibition or anoikis, for example, and how such mechanisms constrain growth rates and spatial patterns. While one could use our conditional logic apparatus to exploit an as yet unimplemented local-density predicate to crudely model constrained growth, Plank, *et al* argue that since lattice-based methods implicitly assume uniform density, among other limitations, properly modeling crowdedness requires a lattice-free setting [57]. In a similar vein, lattice-based simulation, specifically the discrete nature of fitness-based cell replacement, could introduce numerical artifacts that obscure biological interpretation of simulation results, as we discussed above in the results of the metabolic symbiosis simulation.

Second, we may wish to explicitly model individual cell migration. Our simulation presently uses a simple statistical mechanism to implement fitness-based local cell type regional takeover. As mentioned earlier, this implies that individual cells do not possess a unique identity. While distinct cell types can respond to local concentrations, and locally adapt to their environment using their conditional logic, they do so in a manner that ignores their individuality. In other words, our world is lattice-state-centric rather than cell-identity-centric. In the end, this may be too abstract a setting to properly model individual cell migration in a way that can be configured by its own set of parameters. This area has a broad literature [39–45, 50, 58]. Without individual cell identification, it is difficult to determine, for example, whether the local population of hypoxic cells near a vessel is indeed a stable population (infrequent replacement) or the result of dynamic, steady-state replacement. Thus, the relationship between cell migration and emerging local, stable regions of chronic hypoxia is presently unclear to us, but may prove worth exploring.

Third, cell sorting is accomplished by cell migration [58], and cell migration in turn depends on cell-cell adhesion, among other biophysical factors; thus we may wish to explicitly model cell-cell adhesion. Graner and Glazier created the cellular Potts model to simulate cell sorting [41, 42]; since then, others have applied the cellular Potts model to study tumor growth, invasion, and evolution [43], angiogenesis [45], and to create multiscale models of tumor cell growth and invasion that use biophysics simulation frameworks like CompuCell3D and Bionetsolver [44]. Despite the longterm degenerate behavior of the cellular Potts model and the modeling limitations imposed by the central role of surface fluctuations [47], efforts to map the parameters of the model formalism to physical and biological properties of cells suggest it to be a powerful tool for investigating a large range of biological questions [46]. In addition, the cellular Potts model has been extended and enhanced in useful ways, for example: a parallel processing implementation that can efficiently simulate a population of 10^7^ or more cells [48]; and a node-based version that can be implemented on any given domain, so long as it is a proper discretization (regular or irregular, fixed or time-evolving), and can easily interface with continuous mechanics or fluid dynamics models [49]. Beginning with Sulsky and Childress [59], cell-centered Voronoi models of cell sorting have been used to study cell tissue dynamics [60] and estimate the strength of cell-cell interactions under the differential adhesion hypothesis [61]. Cellular Potts models and Voronoi tessellation models explicitly represent cell-cell adhesion in their formulation, and so would likely be better settings to investigate metabolic symbiosis, and determine whether or not the stable cellular striations we observed arise from realistic biological assumptions as regards cell-cell adhesion. Since the broader focus of our study is not on subcellular-scale phenomena, but rather on phenomena emerging from whole cells’ consumption and release behaviors, we chose to model each cell as a point source of diffusion and collection, and thus leave the explicit modeling of cell-cell adhesion and cell sorting for a future study.

To balance matters, let us appeal to parsimony. Although each of these areas of exploration merit consideration, in the scope and context of modeling emerging local, stable regions of chronic hypoxia, our present minimal model already captures some of the salient spatial and dynamic features of the phenomenon.

### Embedding the simulator

In this article, we present a simulator that executes a specified situation exactly once. Because cell reproduction events in the simulation are stochastic, then for any given situation, one would need to run the simulator a number of times until one were confident the results did or did not converge upon some well characterized, recognizable phenomenon. Then one would want to explore nearby situations in the parameter space to quantify the variation of observables (e.g., cell migration speed, population sizes, fitness, structural properties like clustering, connectedness, self-similarity, etc.) Here we sketch how one might embed the simulator in a larger system to meet this challenge in an automated fashion.

The nature and extent of the simulation parameter space derives from the algorithmic specification given above. A number of parameters define any given simulation, those that specify initial conditions and those that specify the entities that operate in the simulation. Initial condition parameters specify: the initial positions in the 3D lattice of the cells of various types; the initial and basal upper- and lower-bound concentrations of the various particle types; and the delay time indicating when to place the cells in their initial positions. Operational parameters specify: each particle type’s diffusion rate; the consumption and release rates and impact factors of each cell type for each particle type; whether each cell type is replaceable and whether its reproductive; and the conditional behaviors for each cell type. Together, these constitute a high dimensional parameter space. Each point in this parameter space is then a full specification to simulate some biological system, and could be represented by a vector input to the simulator.

Let us assume in the absence of simplifying factors or expert knowledge of the biology, each parameter should be modeled as a random variable having a uniform, independent probability distribution. We could then sample the large parameter space using a vanilla Monte Carlo algorithm [62] and accumulate families of nearby solutions. Or we could attempt to make this process more efficient by learning from each sample’s truth outcome if it is in {0, 1}, or branching-and-bounding sampled subspaces where the sample values are in [0, 1]. In this way, one could develop adaptive Monte Carlo methods that, for example, employ boosting of the independent probability distributions upon successful samples, or alternatively, constrained random walks around successful samples. Further, we could adapt the traditional branch-and-bound algorithm [63, 64], so that instead of systematically exploring a subspace of problems, we employ constrained Monte Carlo sampling of each subspace.

Our model (simulation), once specified by a coordinate from the high dimensional parameter space, is still stochastic, as mentioned above, owing to the cells’ manner of probabilistic reproduction. Suppose a feature integrator-detector is embedded in the simulator that decides when the formal characterization of, say, hypoxia—be it a spatiotemporal logical proposition, *φ*, or a learned functional form, *f*—is satisfied. It will then enable the simulator to return a value in {0, 1} or [0, 1], respectively, where the first is a the evaluation of a logical proposition, and the second is a normalized similarity score. Thus, depending on the detection scheme being implemented, the simulator’s outcome can be modeled as either a Bernoulli random variable or a numerical random variable. The first case is the province of statistical model checking. Jha, *et al* [65] gives a provably efficient Bayesian statistical model checking algorithm that would work. It runs the simulator some bounded number of times until enough confidence accrues to the null hypothesis (the simulator satisfies, within some bounded probability, the spatiotemporal logical proposition describing hypoxia), or its alternative hypothesis (it does not). This verdict constitutes the Bayesian-tested outcome of the simulation with respect to satisfying *φ*. In a similar, and perhaps trivial, sense, the numerical [0, 1] outcome of the simulation should be tested repeatedly until some threshold on the numerical stability of its mean value is surpassed. Here a simple approach could be to examine its mean and standard deviation, and apply a threshold to its $CV=\frac{\sigma }{\mu }$.

**Fig 16:**
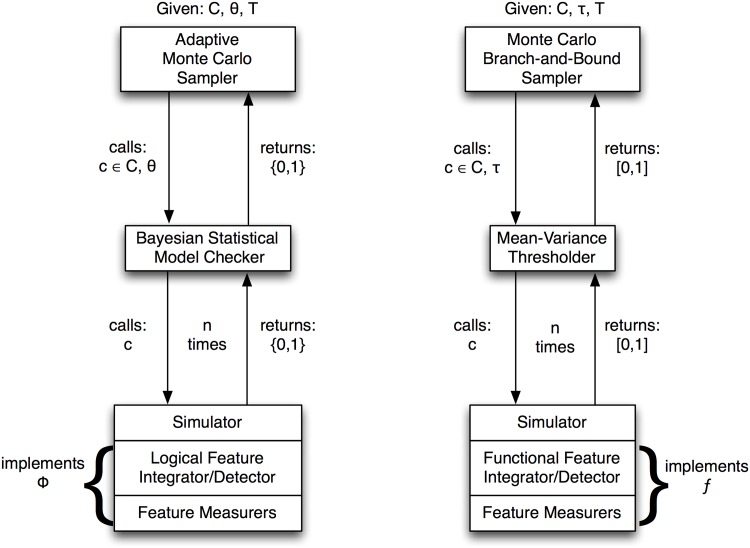
Run-time view of simulator control. Here we show the two possible execution paths of the hypothetical system we propose. These correspond to the choice of whether to use a logical or functional characterization/detection scheme. In the logical scheme, we assume three global parameters: the configuration parameter space, *C*, a probability threshold, *θ*, and a simulation time threshold, *T*. The adaptive Monte Carlo sampling method selects a *c* ∈ *C* and calls the Bayesian statistical model checker with *c* and *θ*. This then calls the simulator with *c*, thereby configuring the simulator for an execution. The simulator embeds the logical feature integrator-detector and feature measurers, together which implement *φ*. The simulator either halts early, returning 1, or runs its full course (to time *T*), returning 0. Depending on this outcome, the Bayesian statistical model checker decides whether or not to execute another simulation of *c*. Once it halts, the Bayesian statistical model checker returns its binary verdict. In the functional scheme, we assume three global parameters: the configuration parameter space, *C*, a coefficient of variation threshold, *τ*, and a simulation time threshold, *T*. The Monte Carlo branch-and-bound sampler selects a *c* ∈ *C* and calls the mean-variance thresholder with *c* and *τ*. This then calls the simulator with *c*, thereby configuring the simulator for an execution. The simulator embeds the functional feature integrator-detector and feature measurers, together which implement *f*. The simulator halts at *T* and returns its “high water mark” normalized similarity score, a real number in [0, 1]. Depending on this outcome, the mean-variance thresholder decides whether or not to execute another simulation of *c*. Once it halts, the mean-variance thresholder returns its binary verdict.

Above we presented two distinct ways of implementing a two-level simulation driver. The top level explores by sampling the high dimensional parameter space, testing a coordinate in that space by passing control down to the lower level that repeatedly runs the specified simulation until a stable outcome is achieved. It then passes the binary verdict up to the top level that records and eventually responds to the coordinate’s computed truth value. We envision such a system would have a run-time design like in Fig 16. We leave this for a future study.

## Supporting Information

S1 Fig Spatial frequency of metabolic symbiosis striations. Each simulation produces a spatial cell occupation map as it evolves and as its final output. To show the consistent spatial periodicity of the striations of *aerobic* and *hypoxic* cells across simulations, we used Matlab’s FFT2 function to transform each resulting occupation map into the frequency domain, then examined the mean FFT2 magnitude over 10 simulations. Notice the two energy loci above and below the center. They are distant from the center due to the sharp boundaries (and thus high spatial frequency information) of the striations. They are vertically oriented from the center because the striations tend to be horizontal and are thus perpendicular to the FFT2 magnitude orientation. And the loci are tightly clustered, indicating the consistent periodicity of striations between simulations.
(TIFF)

S2 Fig Dispersion in the spatial frequency of metabolic symbiosis striations. Standard deviation (SD) in FFT2 magnitude across 10 simulations. The maximum standard deviation is 0.43 times the maximum mean value.
(TIFF)

S3 Fig Noise-to-signal in the spatial frequency of metabolic symbiosis striations. Coefficient of variation (CV) in FFT2 magnitude across 10 simulations. Notice no regions of high noise-to-signal ratio colocate with the two energy loci; rather, the noise appears uniformly distributed across the energy surface.
(TIFF)

S4 Fig Population evolution of metabolic symbiosis. Mean *aerobic* (green) and *hypoxic* (red) populations across 10 simulations. All simulation trajectories are shown (gray).
(TIFF)

S5 Fig Dispersion in the population evolution of metabolic symbiosis. Standard deviation (SD) in *aerobic* (green) and *hypoxic* (red) population sizes across 10 simulations. Notice the SDs are identical for *hypoxic* and *aerobic* populations—green is overlaid atop red—due to their zero-sum relationship; a gain in one population is precisely the loss in the other, and vice-versa. The maximum SD is 0.12 times the maximum mean value.
(TIFF)

S6 Fig Noise-to-signal in the population evolution of metabolic symbiosis. Coefficient of variation (CV) in *aerobic* (green) and *hypoxic* (red) population sizes across 10 simulations. Unlike their respective standard deviations, the populations have differing CVs since their respective denominators (mean population sizes) differ. The maximum CV is 0.12.
(TIFF)

S7 Fig Population evolution of tumor-stroma signaling. Mean *tumor* (orange) population across 10 simulations. All simulation trajectories are shown (gray). Notice the onset of tumor growth varies by 120 time units (due to the random positioning of reciprocally-signaling *fibroblast* cells, and thus the onset of the positive growth feedback), but once growth onset occurs, the shape and slope of that growth is similar.
(TIFF)

S8 Fig Dispersion in the population evolution of tumor-stroma signaling. Standard deviation (SD) in *tumor* (orange) population size across 10 simulations. The apparently large SD values are due to the variation in growth onset times, as can be seen in the simulation trajectories, and trying to fit them to a unimodal Gaussian distribution.
(TIFF)

S9 Fig Noise-to-signal in the population evolution of tumor-stroma signaling. Coefficient of variation (CV) in *tumor* (orange) population size across 10 simulations. The apparently large CV values are due to the variation in growth onset times, as can be seen in the simulation trajectories, and trying to fit them to a unimodal Gaussian distribution.
(TIFF)

S10 Fig Population evolution of stable local chronic hypoxia with many vessels (2D). Mean *viable* (red), *hypoxic* (green), and *necrotic* (orange) populations across 10 simulations. All simulation trajectories are shown (gray).
(TIFF)

S11 Fig Dispersion in the population evolution of stable local chronic hypoxia with many vessels (2D). Standard deviation (SD) in *viable* (red), *hypoxic* (green), and *necrotic* (orange) population sizes across 10 simulations. The apparently large and growing SD values after time 150 is due to the randomly placed vessels causing differing patterns of growth and decay in the *viable* and *hypoxic* populations.
(TIFF)

S12 Fig Noise-to-signal in the population evolution of stable local chronic hypoxia with many vessels (2D). Coefficient of variation (CV) in *viable* (red), *hypoxic* (green), and *necrotic* (orange) population sizes across 10 simulations. Despite apparently large and growing SD values after time 150, we see the corresponding CV values drop sharply and remain low.
(TIFF)

S13 Fig Population evolution of stable local chronic hypoxia with many vessels (3D). Mean *viable* (red), *hypoxic* (green), and *necrotic* (orange) populations across 10 simulations. All simulation trajectories are shown (gray).
(TIFF)

S14 Fig Dispersion in the population evolution of stable local chronic hypoxia with many vessels (3D). Standard deviation (SD) in *viable* (red), *hypoxic* (green), and *necrotic* (orange) population sizes across 10 simulations. After the successive fluctuations in *viable* then *hypoxic* then *necrotic* populations (after time 150), SD values drop sharply, as we expect from stable co-existing populations at nearly identical sizes across simulations.
(TIFF)

S15 Fig Noise-to-signal in the population evolution of stable local chronic hypoxia with many vessels (3D). Coefficient of variation (CV) in *viable* (red), *hypoxic* (green), and *necrotic* (orange) population sizes across 10 simulations. After the successive fluctuations in *viable* then *hypoxic* then *necrotic* populations (after time 150), CV values drop sharply, as we expect from stable co-existing populations at nearly identical sizes across simulations. The higher CV for the *viable* population size is due to the denominator (mean population size) fluctuating near zero consistently across simulations.
(TIFF)

S1 Table (TEX)
