# Supplementary figures and images for: Simulating Heterogeneous Tumor Cell Populations
Source: PLoS One. 2016 Dec 28;11(12):e0168984. doi: 10.1371/journal.pone.0168984 (PMC5193460; doi:10.1371/journal.pone.0168984)

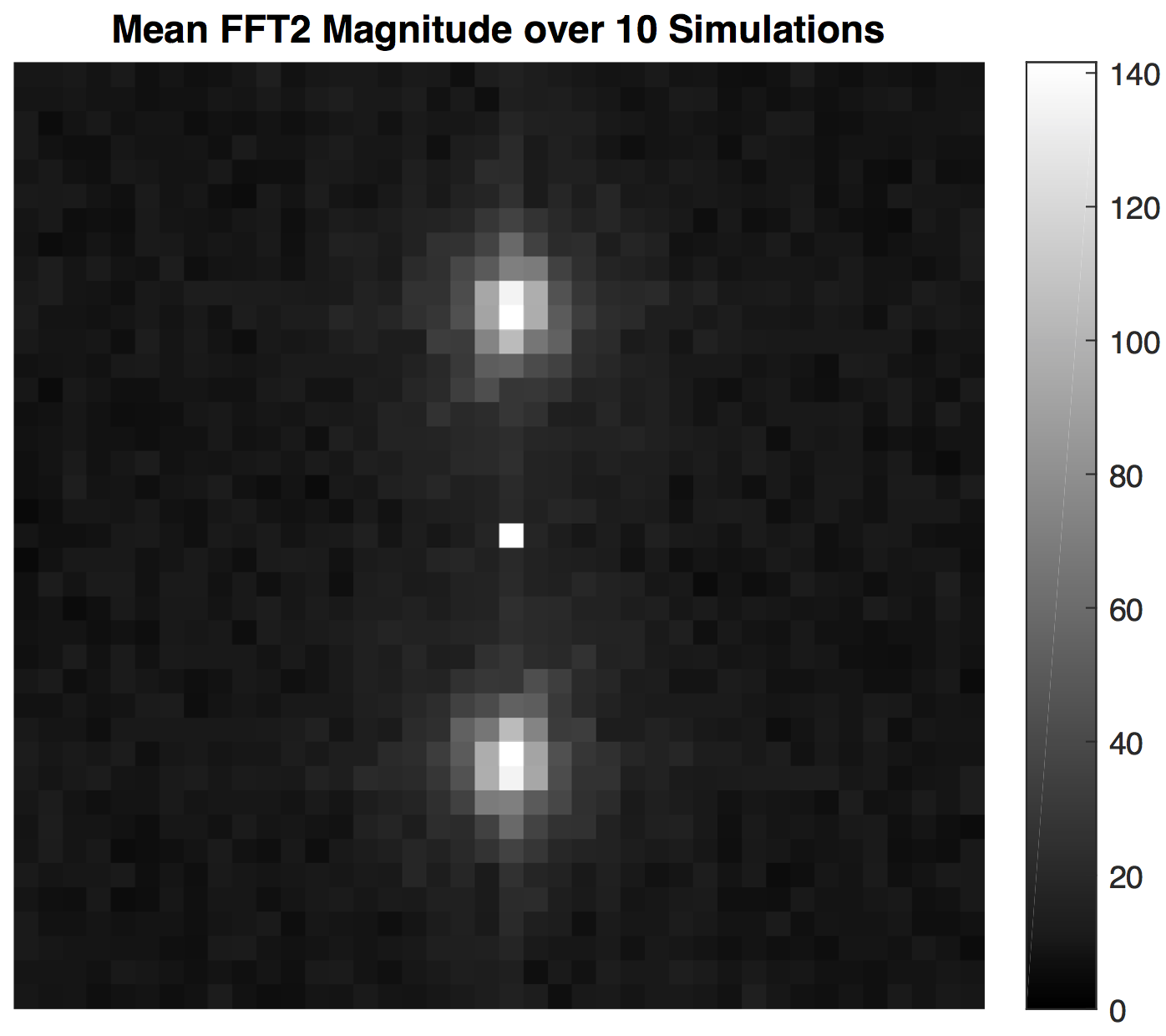

Supplement: S1 Fig — Each simulation produces a spatial cell occupation map as it evolves and as its final output. To show the consistent spatial periodicity of the striations of aerobic and hypoxic cells across simulations, we used Matlab’s FFT2 function to transform each resulting occupation map into the frequency domain, then examined the mean FFT2 magnitude over 10 simulations. Notice the two energy loci above and below the center. They are distant from the center due to the sharp boundaries (and thus high spatial frequency information) of the striations. They are vertically oriented from the center because the striations tend to be horizontal and are thus perpendicular to the FFT2 magnitude orientation. And the loci are tightly clustered, indicating the consistent periodicity of striations between simulations. (TIFF) [file pone.0168984.s001.tiff]

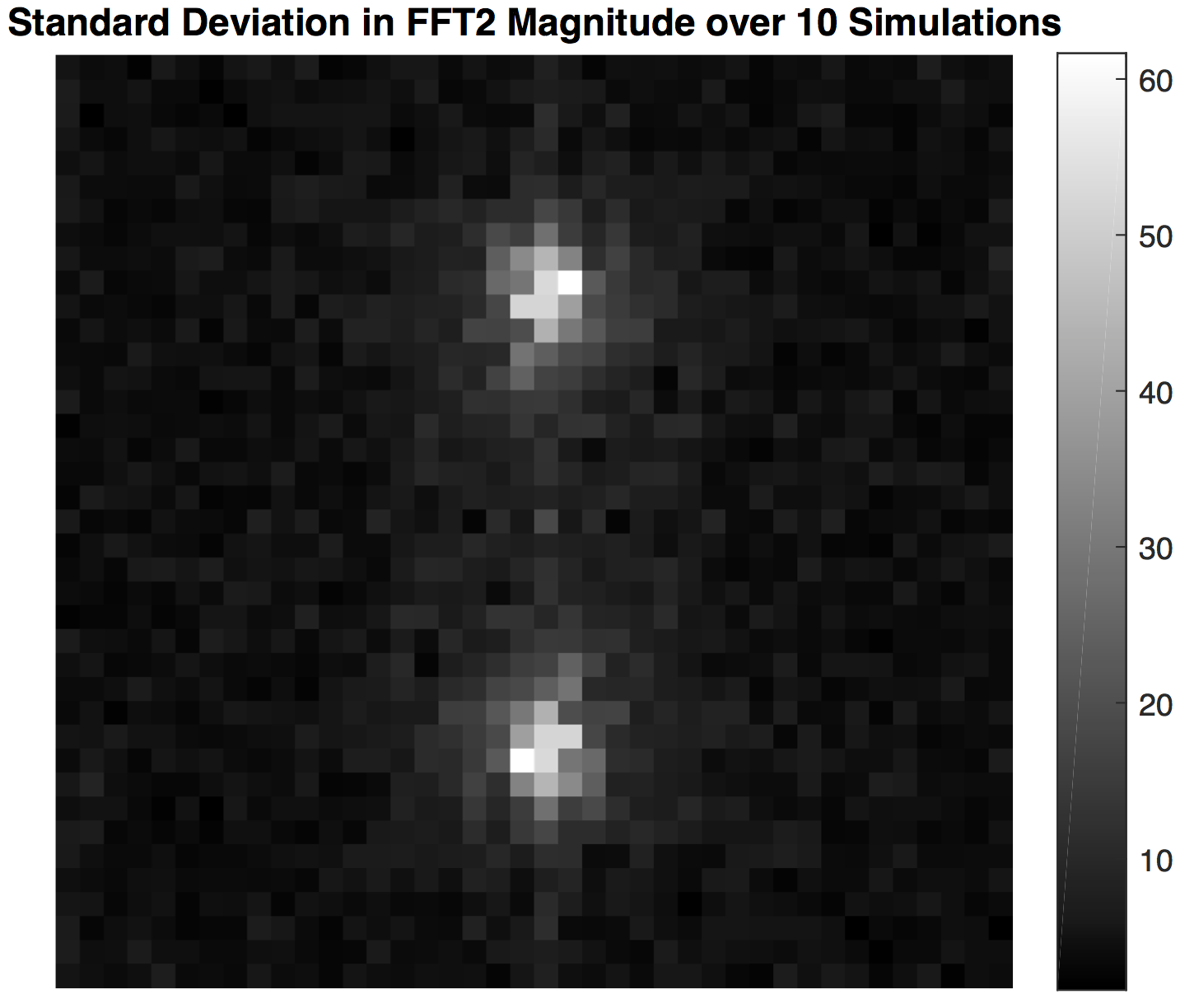

Supplement: S2 Fig — Standard deviation (SD) in FFT2 magnitude across 10 simulations. The maximum standard deviation is 0.43 times the maximum mean value. (TIFF) [file pone.0168984.s002.tiff]

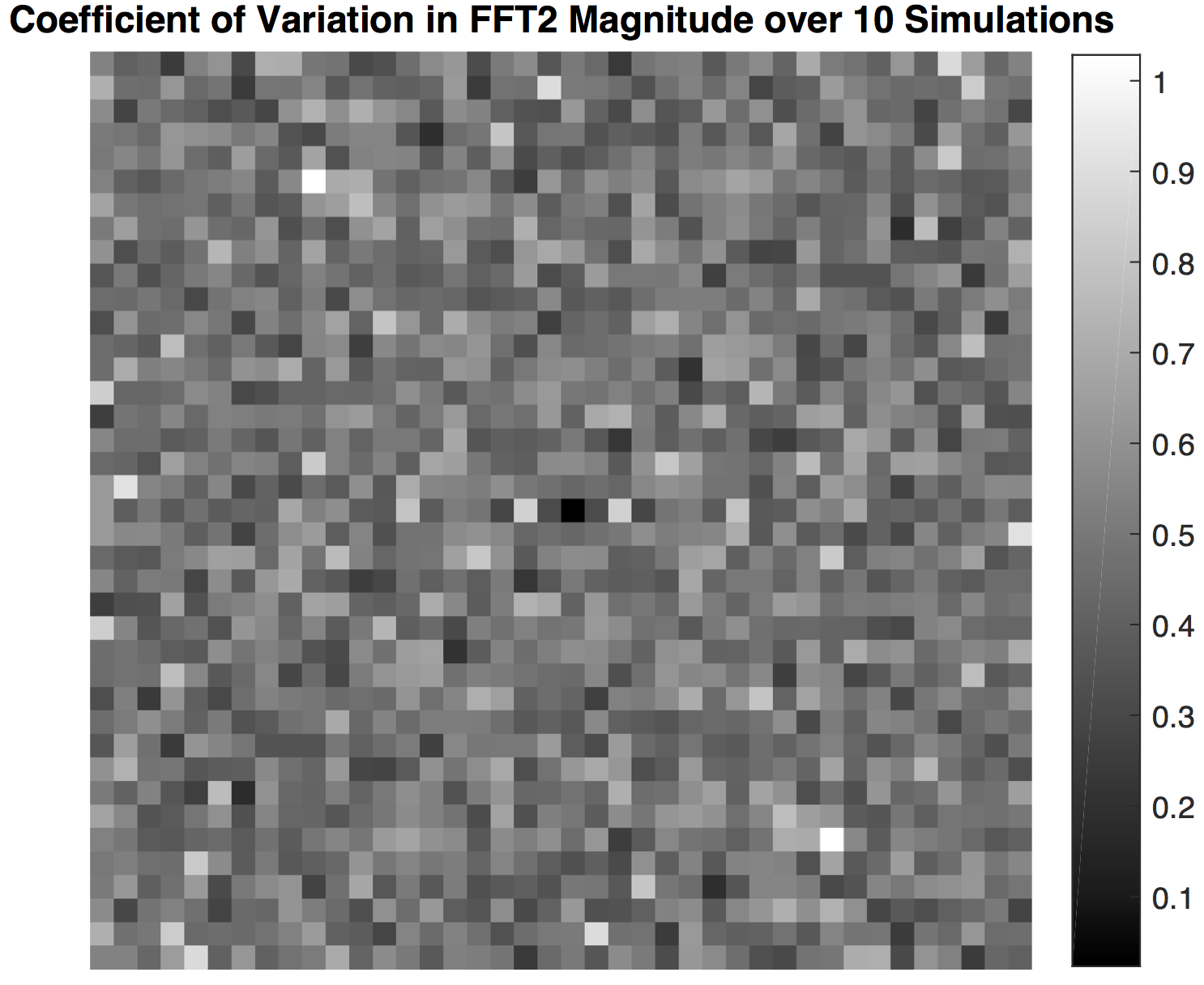

Supplement: S3 Fig — Coefficient of variation (CV) in FFT2 magnitude across 10 simulations. Notice no regions of high noise-to-signal ratio colocate with the two energy loci; rather, the noise appears uniformly distributed across the energy surface. (TIFF) [file pone.0168984.s003.tiff]

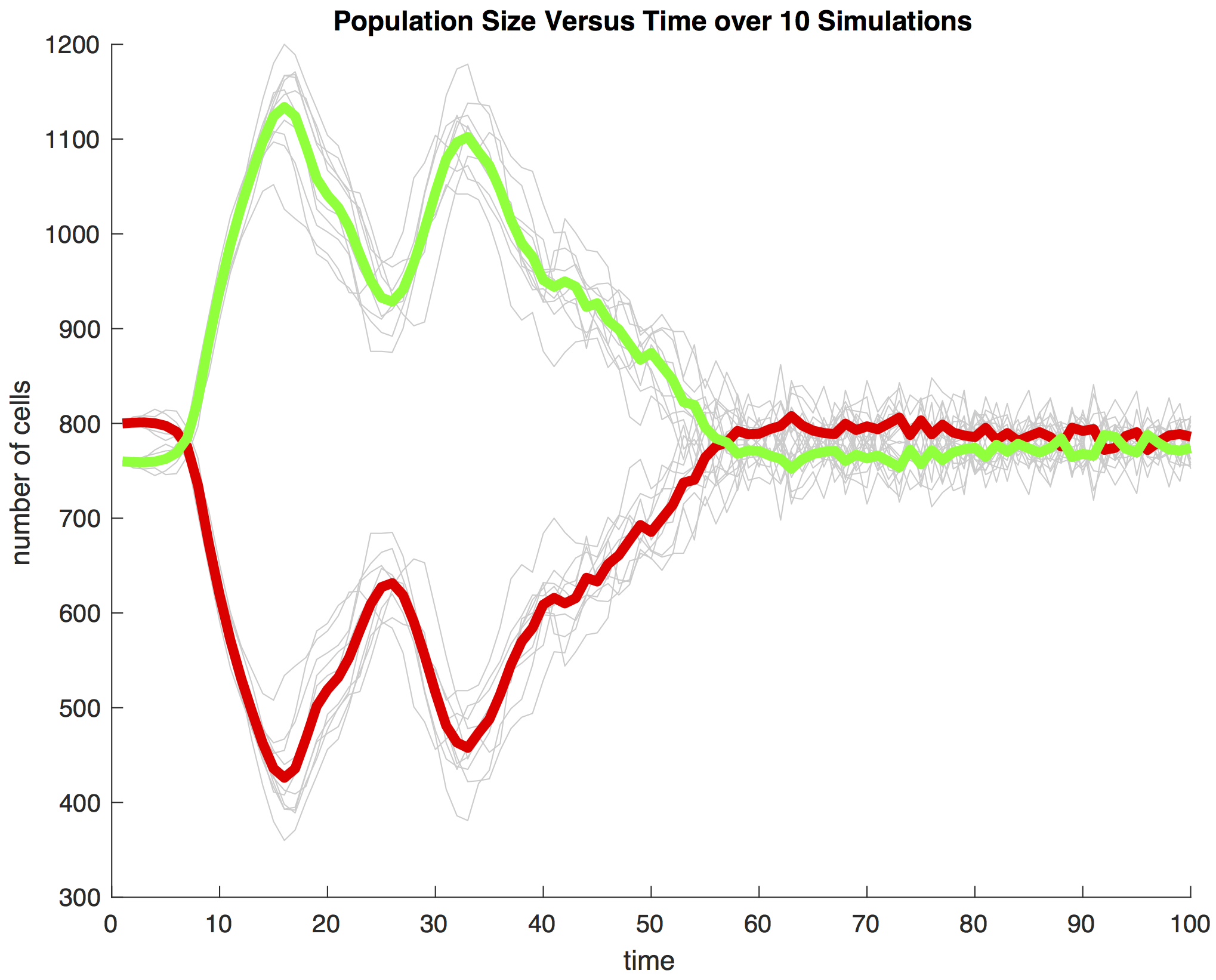

Supplement: S4 Fig — Mean aerobic (green) and hypoxic (red) populations across 10 simulations. All simulation trajectories are shown (gray). (TIFF) [file pone.0168984.s004.tiff]

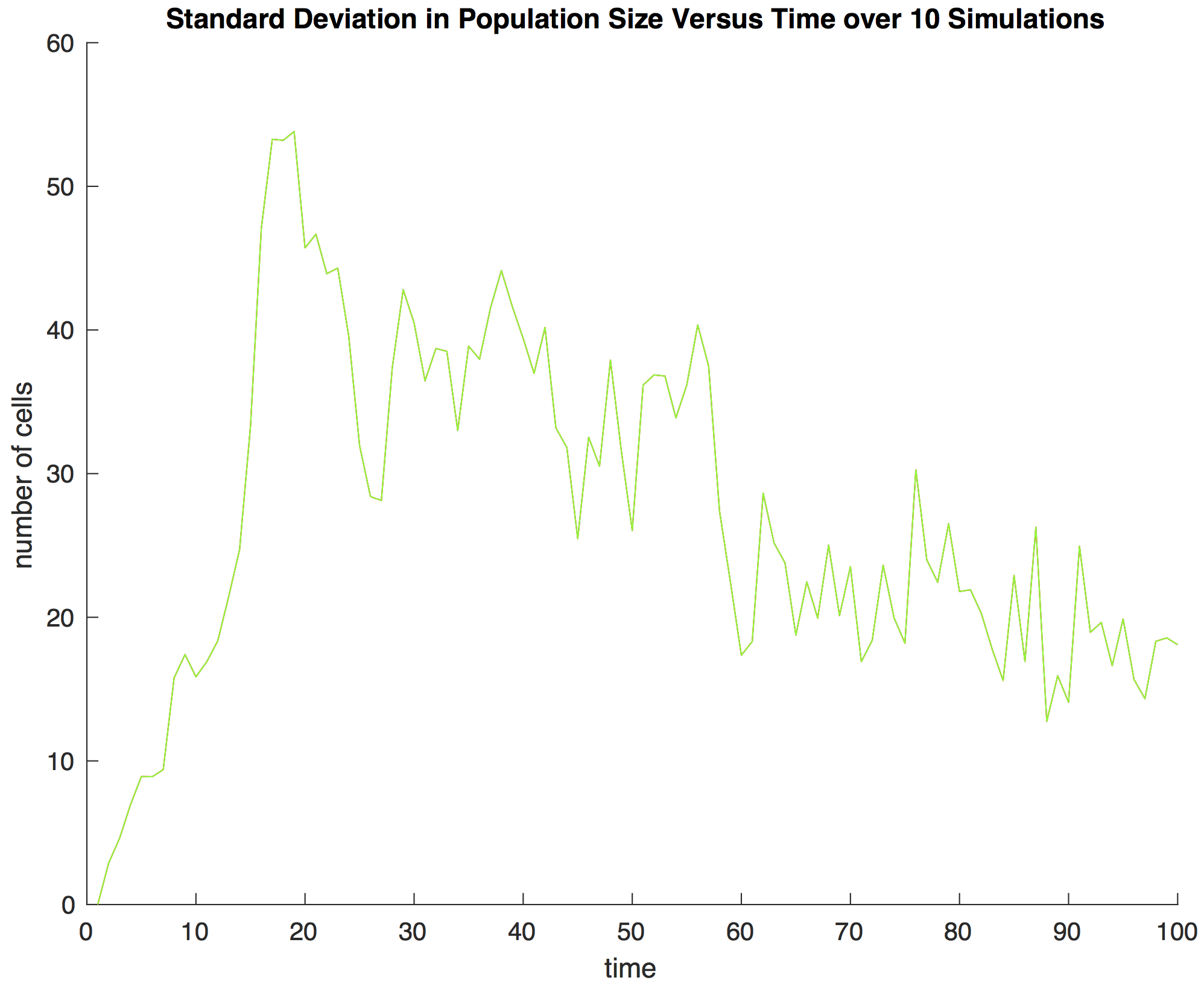

Supplement: S5 Fig — Standard deviation (SD) in aerobic (green) and hypoxic (red) population sizes across 10 simulations. Notice the SDs are identical for hypoxic and aerobic populations—green is overlaid atop red—due to their zero-sum relationship; a gain in one population is precisely the loss in the other, and vice-versa. The maximum SD is 0.12 times the maximum mean value. (TIFF) [file pone.0168984.s005.tiff]

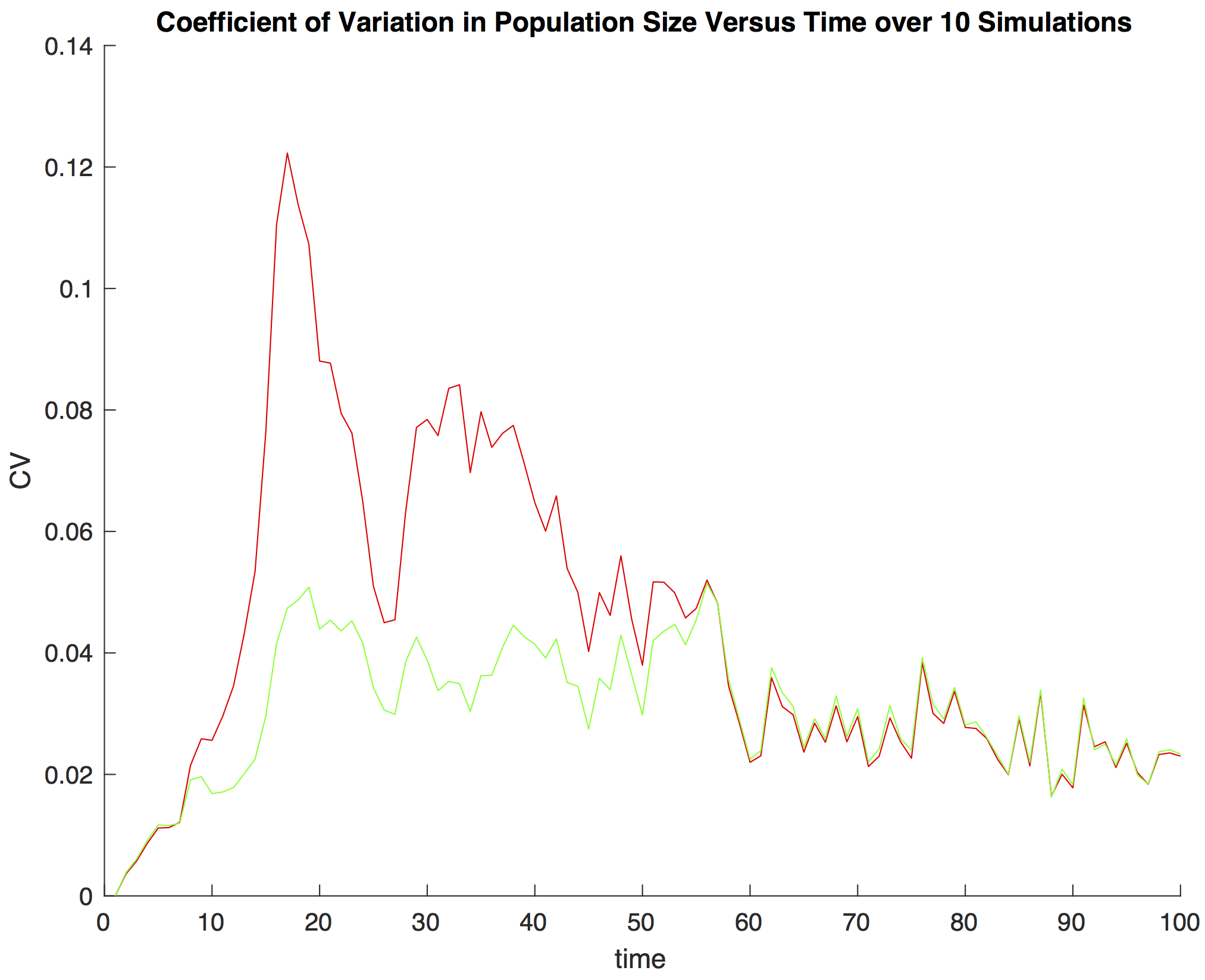

Supplement: S6 Fig — Coefficient of variation (CV) in aerobic (green) and hypoxic (red) population sizes across 10 simulations. Unlike their respective standard deviations, the populations have differing CVs since their respective denominators (mean population sizes) differ. The maximum CV is 0.12. (TIFF) [file pone.0168984.s006.tiff]

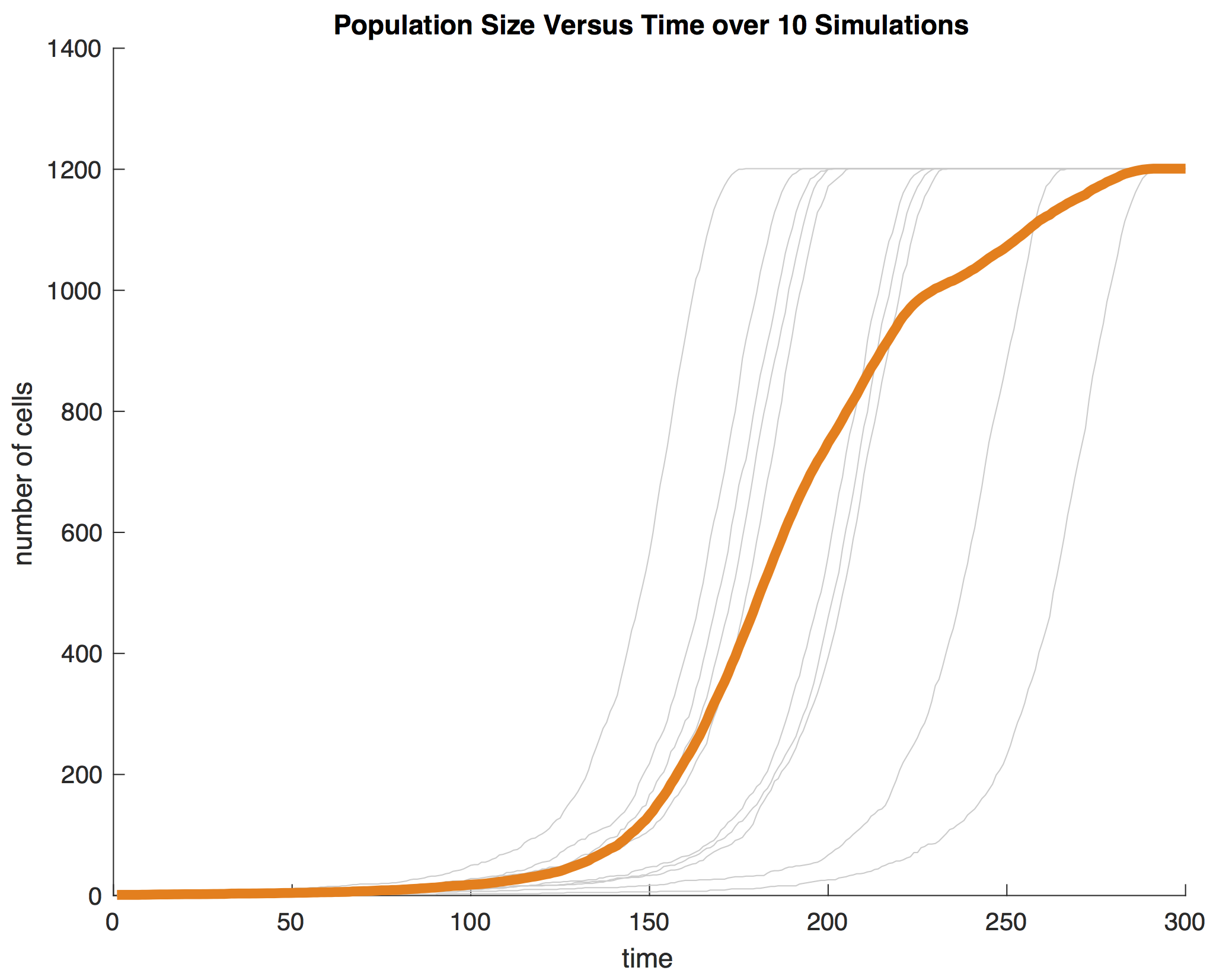

Supplement: S7 Fig — Mean tumor (orange) population across 10 simulations. All simulation trajectories are shown (gray). Notice the onset of tumor growth varies by 120 time units (due to the random positioning of reciprocally-signaling fibroblast cells, and thus the onset of the positive growth feedback), but once growth onset occurs, the shape and slope of that growth is similar. (TIFF) [file pone.0168984.s007.tiff]

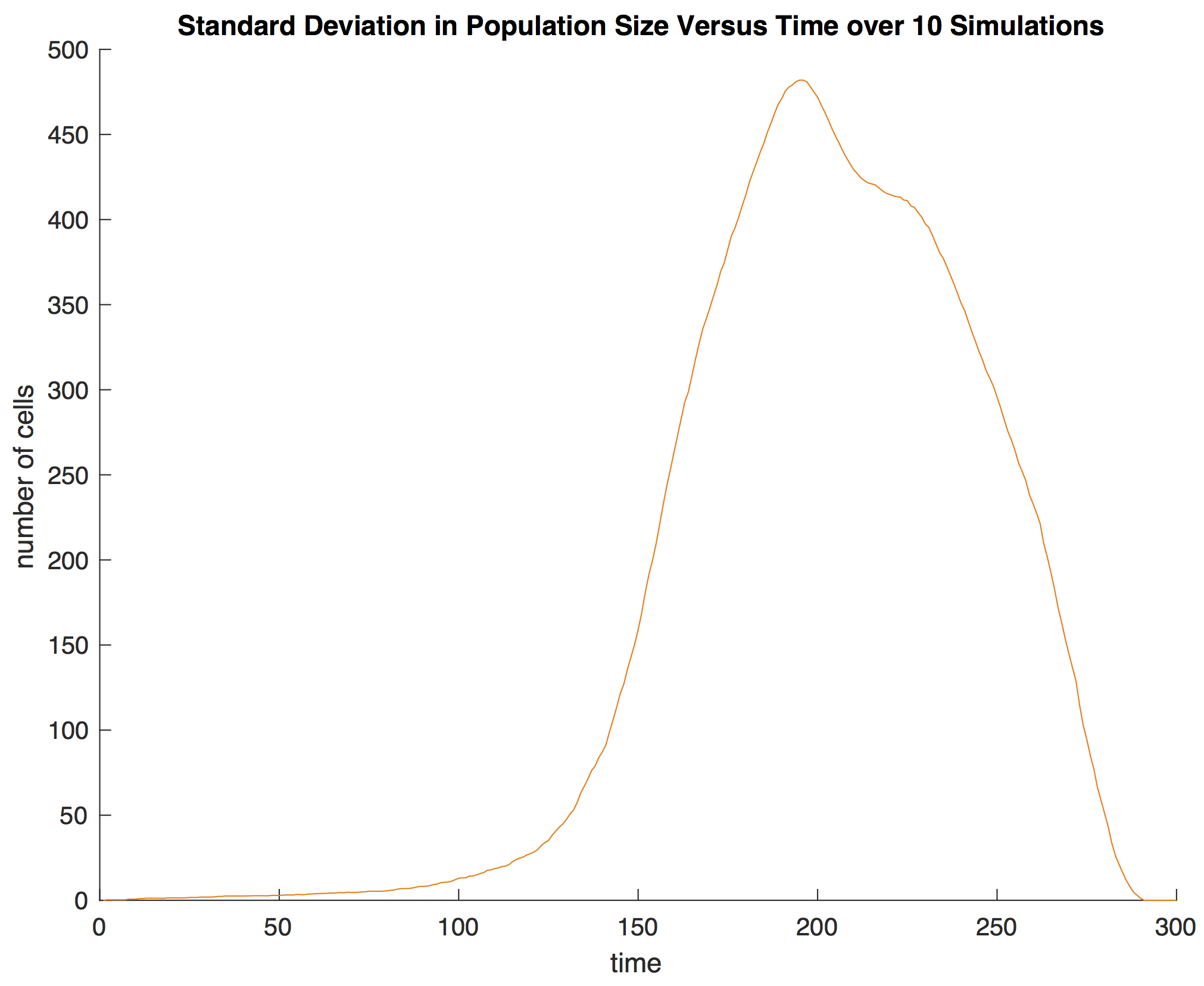

Supplement: S8 Fig — Standard deviation (SD) in tumor (orange) population size across 10 simulations. The apparently large SD values are due to the variation in growth onset times, as can be seen in the simulation trajectories, and trying to fit them to a unimodal Gaussian distribution. (TIFF) [file pone.0168984.s008.tiff]

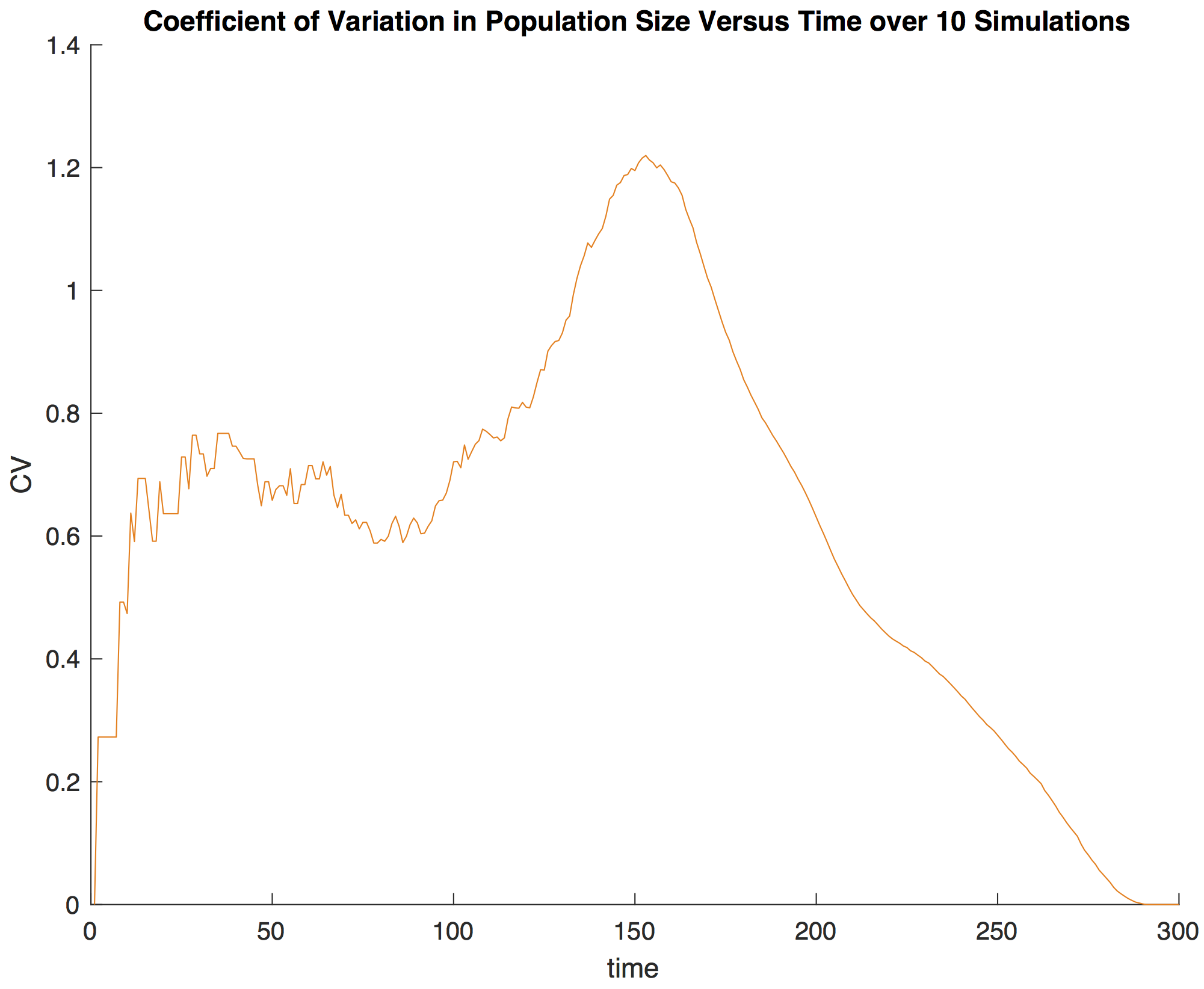

Supplement: S9 Fig — Coefficient of variation (CV) in tumor (orange) population size across 10 simulations. The apparently large CV values are due to the variation in growth onset times, as can be seen in the simulation trajectories, and trying to fit them to a unimodal Gaussian distribution. (TIFF) [file pone.0168984.s009.tiff]

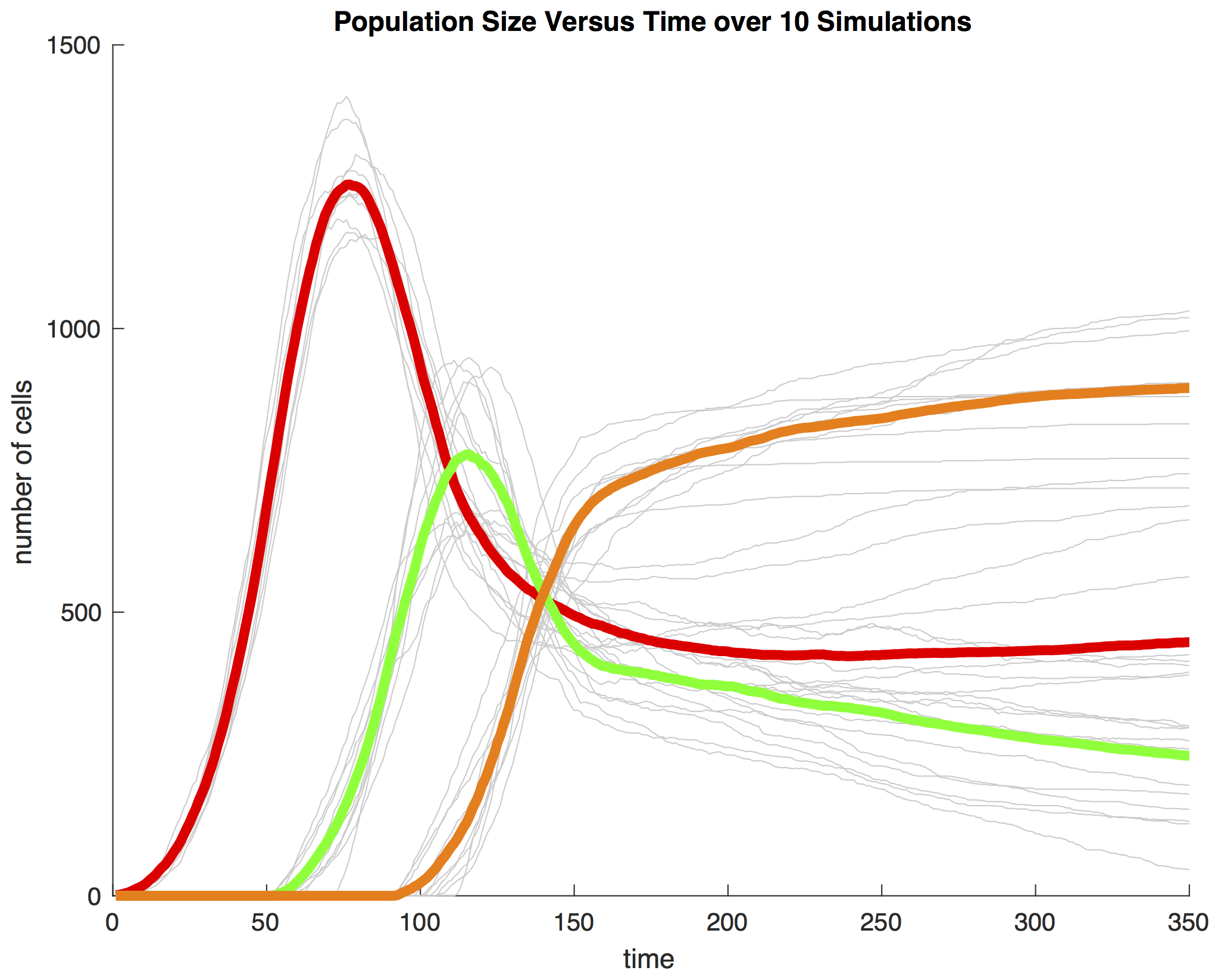

Supplement: S10 Fig — Mean viable (red), hypoxic (green), and necrotic (orange) populations across 10 simulations. All simulation trajectories are shown (gray). (TIFF) [file pone.0168984.s010.tiff]

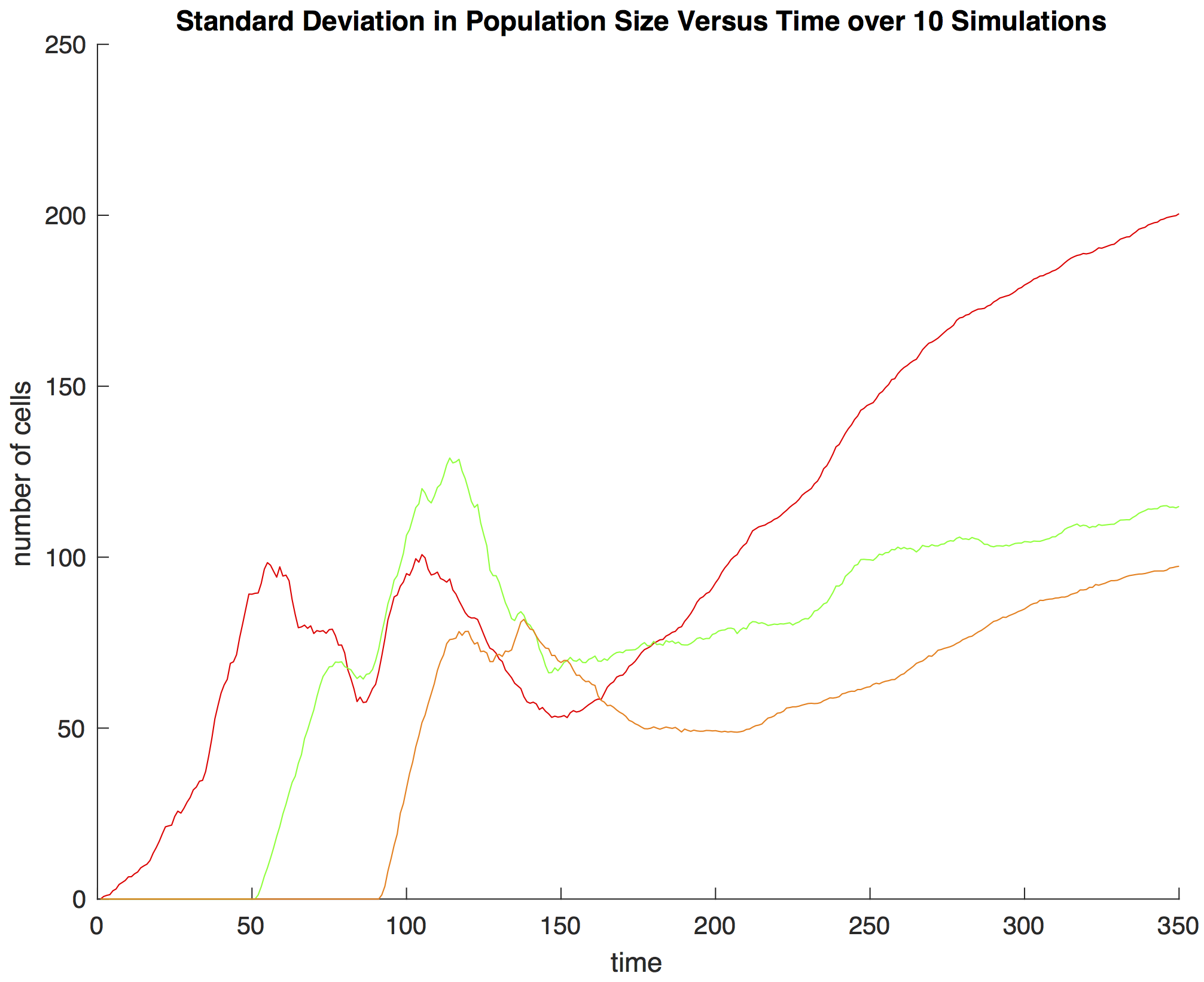

Supplement: S11 Fig — Standard deviation (SD) in viable (red), hypoxic (green), and necrotic (orange) population sizes across 10 simulations. The apparently large and growing SD values after time 150 is due to the randomly placed vessels causing differing patterns of growth and decay in the viable and hypoxic populations. (TIFF) [file pone.0168984.s011.tiff]

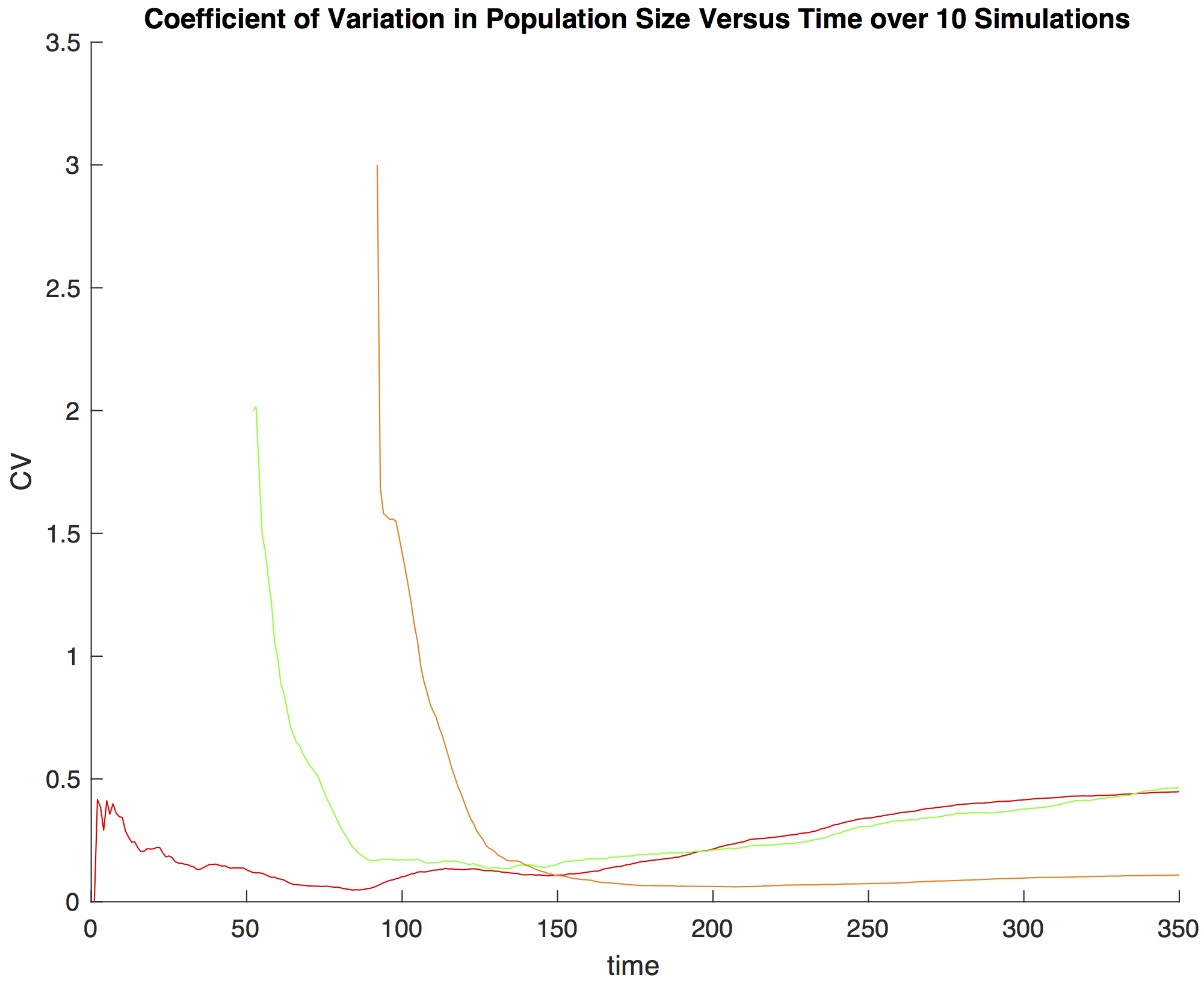

Supplement: S12 Fig — Coefficient of variation (CV) in viable (red), hypoxic (green), and necrotic (orange) population sizes across 10 simulations. Despite apparently large and growing SD values after time 150, we see the corresponding CV values drop sharply and remain low. (TIFF) [file pone.0168984.s012.tiff]

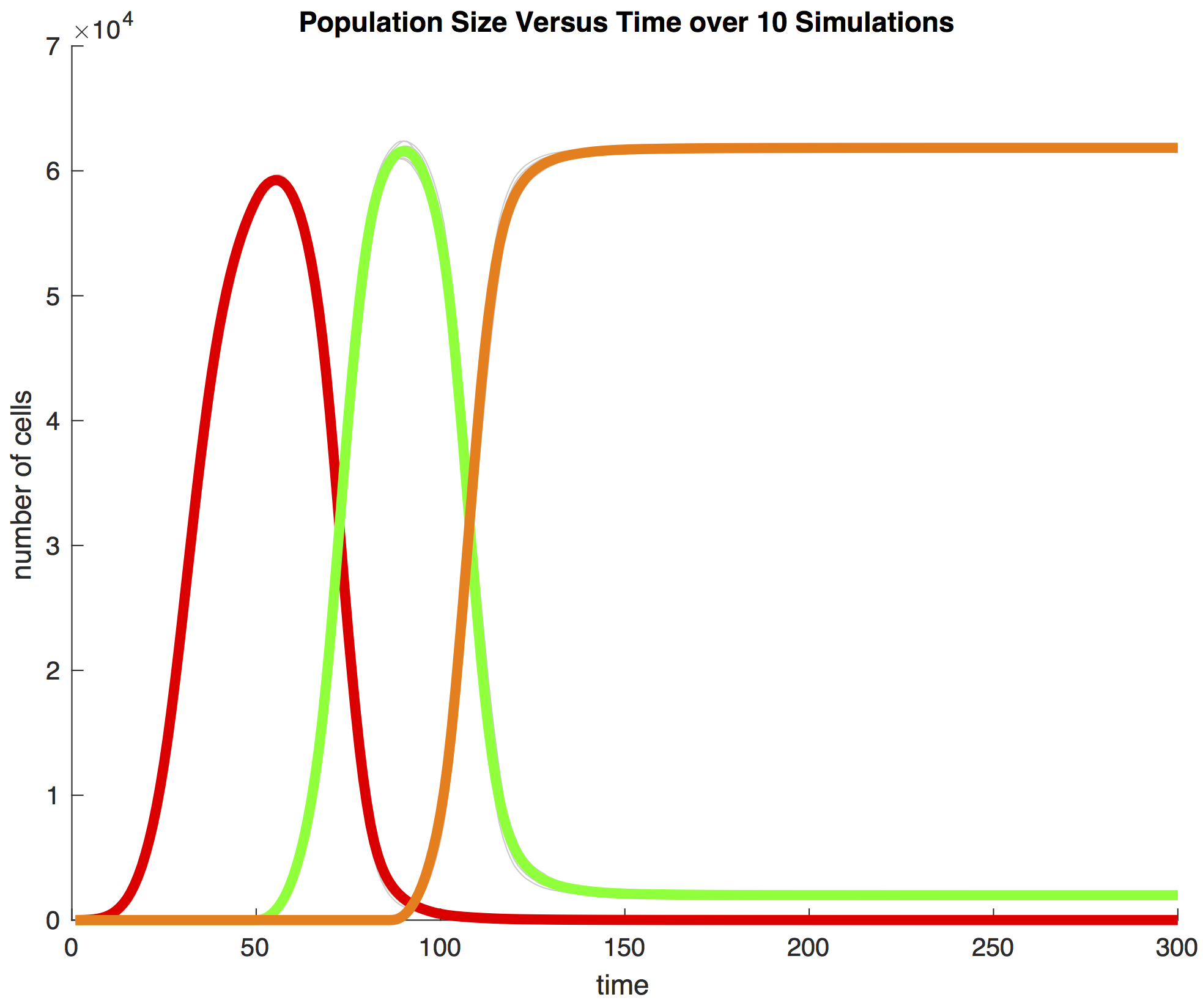

Supplement: S13 Fig — Mean viable (red), hypoxic (green), and necrotic (orange) populations across 10 simulations. All simulation trajectories are shown (gray). (TIFF) [file pone.0168984.s013.tiff]

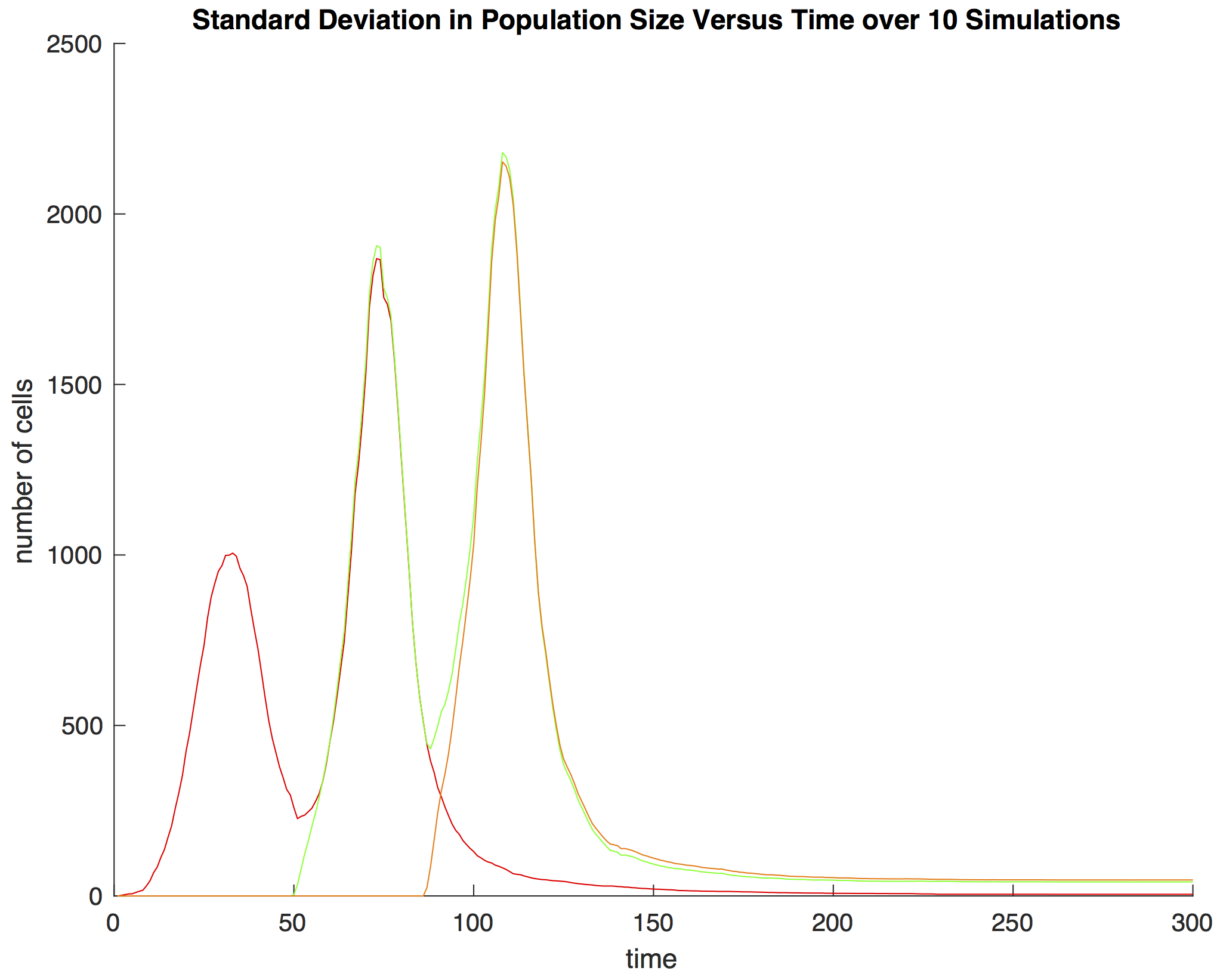

Supplement: S14 Fig — Standard deviation (SD) in viable (red), hypoxic (green), and necrotic (orange) population sizes across 10 simulations. After the successive fluctuations in viable then hypoxic then necrotic populations (after time 150), SD values drop sharply, as we expect from stable co-existing populations at nearly identical sizes across simulations. (TIFF) [file pone.0168984.s014.tiff]

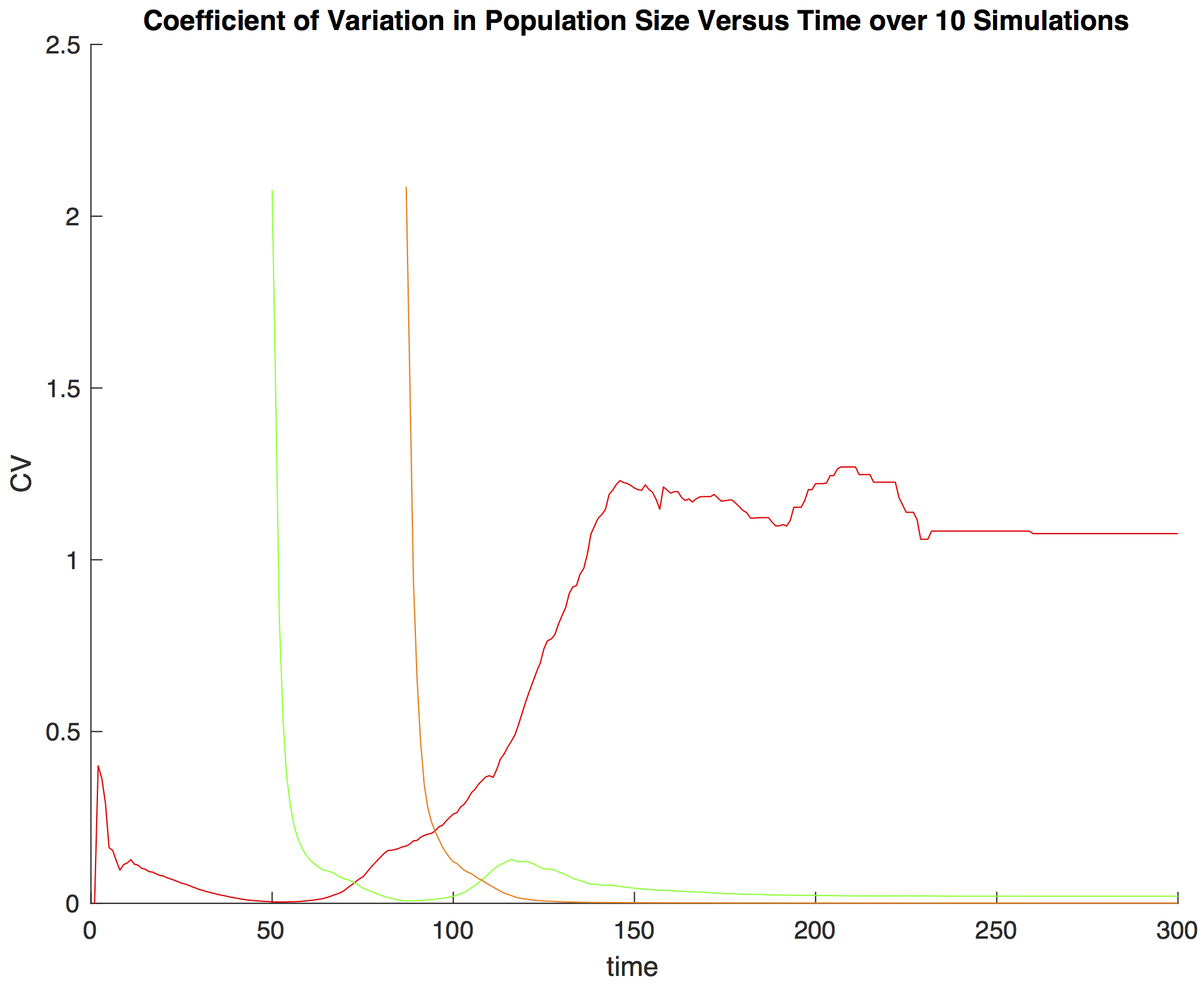

Supplement: S15 Fig — Coefficient of variation (CV) in viable (red), hypoxic (green), and necrotic (orange) population sizes across 10 simulations. After the successive fluctuations in viable then hypoxic then necrotic populations (after time 150), CV values drop sharply, as we expect from stable co-existing populations at nearly identical sizes across simulations. The higher CV for the viable population size is due to the denominator (mean population size) fluctuating near zero consistently across simulations. (TIFF) [file pone.0168984.s015.tiff]
